# Functionalized Micellar Membranes from Medicinal Mushrooms as Promising Self-Growing Bioscaffolds

**DOI:** 10.3390/polym17172334

**Published:** 2025-08-28

**Authors:** Nika Kučuk, Mateja Primožič, Željko Knez, Maja Leitgeb

**Affiliations:** 1 Faculty of Chemistry and Chemical Engineering, University of Maribor, Smetanova 17, 2000 Maribor, Slovenia; nika.kucuk1@gmail.com (N.K.); mateja.primozic@um.si (M.P.); zeljko.knez@um.si (Ž.K.); 2 Faculty of Medicine, University of Maribor, Taborska ulica 8, 2000 Maribor, Slovenia

**Keywords:** medicinal mushrooms, *Ganoderma lucidum*, *Pleurotus ostreatus*, micellar membranes, functionalization, mango peels, in vitro release, antibacterial

## Abstract

Micellar or mycelial membranes from medicinal mushrooms are self-growing fibrous polymeric biocomposites that are biocompatible, biodegradable, cost-effective, and environmentally friendly. In this study, the cultivation process for the medicinal mushrooms *Ganoderma lucidum* and *Pleurotus ostreatus* has been optimized via submerged cultivation to maximize growth and promote the formation of micellar membranes with high water-absorption capacity. Optimal growth conditions were achieved at an alkaline pH in a medium containing malt extract for *G. lucidum*, while for *P. ostreatus*, these were in a glucose-enriched medium. The hydrophilic underside of the micellar membranes led to a high-water uptake capacity. These membranes exhibited a broad spectrum of functional groups, thermal stability with decomposition temperatures above 260 °C, and a fibrous and porous structure. The micellar membranes from both mushrooms were additionally functionalized with mango peel extract (MPE), resulting in a uniform and gradual release profile, which is an important novelty. They also showed successful antimicrobial activity against *Escherichia coli* and *Staphylococcus aureus* growth. MPE-functionalized micellar membranes are, therefore, innovative biocomposites suitable for various biomedical applications. As they mimic the extracellular matrix of the skin, they are a promising material for tissue engineering, wound healing, and advanced skin materials applications.

## 1. Introduction

In recent years, the development of innovative, environmentally friendly, and sustainable biocomposites for biomedical applications has become increasingly important [1]. Various natural and sustainable resources, such as lignin, cellulose, pectin, protein products, etc., are the main sources for the production of biopolymeric materials. They are environmentally friendly, easily degradable, and renewable biomaterials with many other important properties [2]. However, the main difficulty in obtaining these biopolymeric materials is the high cost of the synthesis and purification process. Moreover, production must be accompanied by a high yield of products [3]. Therefore, a straightforward, economical, less time-consuming, high-yield, and innovative approach is the synthesis of polymeric materials from biological sources such as fungi, especially mushrooms [4,5]. The mycelium of fungi, which consists mainly of cellulose, chitin, and various proteins, is a larger group of interwoven hyphae and forms the vegetative part of the fungus [6]. Research in recent years has shown that mycelium-based materials have the potential for a wide range of functions [7]. However, research has mainly focused on the cultivation of fungi on agricultural waste and the analysis of the whole material, i.e., the mycelium embedded in the substrate, as a promising biocomposite [8]. The advantages of micellar or mycelial composites as bio-based materials are their adaptability to different growth conditions, biodegradability, and cost efficiency [9]. Recent research studies have shown that non compacted foam-like mycelial composites can compete with conventional materials such as expanded polystyrene and other foams or biocomposites such as hempcrete [10,11]. Micellar biomaterials are a potential substitute for less environmentally friendly materials. They are entirely natural and can be composted, minimizing waste, which supports the transition to a circular economy [10].

Therefore, micellar biocomposites derived from medicinal mushrooms are an emerging cost-effective and environmentally friendly material class. As fibrous and self-growing polymeric biocomposites, they have gained great importance due to their economic, sustainability, and material characteristics, which mainly depend on the type of substrate used for their production [12,13]. They are considered biocompatible and biodegradable and are also stable, flexible, flame-retardant, pliable, porous, and fast-growing and have self-healing properties [14].

Mycelium-based materials have demonstrated their potential as composites in a variety of fields, including textiles [9], construction [13], paper [15], and packaging [16], as well as promising applications in the biomedical [17] and cosmetic industries [18]. They have proven to be a new source of sustainable packaging materials, as they can potentially replace conventional packaging materials [16]. Their fibrous structure makes them unique and promising for various biological applications [12]. To date, research on the use of mushroom mycelia for biomedical applications has mainly focused on their extracts and derivatives [19].

In addition to their filamentous structure and high mechanical strength, the presence of bioactive molecules (polysaccharides, polyphenols, carotenoids) in the mycelia of edible mushrooms is an essential feature for the development of smart bioscaffolds for tissue engineering with potential anti-inflammatory and antioxidant effects. Micellar materials thus represent a new platform for wound healing, as they form a three-dimensional, interwoven fiber network that resembles the extracellular matrix of the skin [20]. Ruggeri et al. [20] confirmed, in an in vivo study, that the membranes of *Ganoderma lucidum* and *Pleurotus ostreatus* increase collagen I gene expression. The β-glucans in the mycelia play a key role in stimulating the synthesis of collagen type I and III and thus promote wound healing. On the other hand, there is little research on studying the mycelium without a solid substrate as an independent and porous biomaterial [6,21,22]. Micellar materials can be obtained directly via submerged cultivation in an aqueous environment in a growth medium. There is also no need for the use of toxic solvents or further post-treatment of the membranes [23,24,25,26]. Micellar or mycelial membranes derived from therapeutic mushrooms such as *P. ostreatus* and *G. lucidum* are biocompatible and biodegradable, making them promising biocomposites for applications in tissue engineering and wound healing [22].

Their functionalization with various therapeutic agents (such as extracts, bioactive substances, antibiotics etc.) adds additional value [17]. For example, functionalized micellar membranes with the bioactive substance curcumin have shown good antimicrobial activity and the sustained release of curcumin from the micellar matrix at the wound site [17].

On the other hand, agro-industrial waste from the fruit industry is contributing to a growing environmental problem through improper disposal. However, mango by-products, especially peels [27], represent an important source of bioactive substances such as ellagic acid, gallic acid, catechin and mangiferin, which have important health-promoting properties. Their synergistic effects offer a comprehensive approach for the prevention and treatment of diseases in areas such as cancer, cardiovascular health, inflammation, and neuroprotection, making the mango peel extract a promising candidate for the enrichment of micellar membranes for various pharmaceutical developments, especially in the field of tissue engineering and wound healing. In addition, their further use could reduce waste and negative environmental impacts. There is a lack of studies on the production and characterization of micellar membranes. At the same time, functionalization with natural extracts has not yet been reported, which represents the development of potential therapeutic materials with high added value.

The aim of the study was to obtain micellar membranes from the medicinal mushrooms *G. lucidum* and *P. ostreatus* via submerged cultivation. The cultivation parameters (composition and pH of the growth medium and the cultivation time) were optimized to achieve the highest growth and formation of micellar membranes, as well as a high-water-uptake capacity. The morphological characteristics and the structure of the hyphae and pores were investigated. A hydrodynamic characterization was also performed, including the hydrophilicity/hydrophobicity of the membrane surfaces and the swelling kinetics. In addition, the micellar membranes have been functionalized with mango peel extract (MPE) to increase the therapeutic potential of the micellar membranes, as MPE has many health benefits. The incorporation efficiency (IE) and loading capacity (LC) were determined, and the in vitro release of MPE from the membranes was also monitored. The antimicrobial activity of the MPE-loaded micellar membranes was validated using a modified disk diffusion method and a plate count method. The antimicrobial potential was tested against the growth of Gram-negative *Escherichia coli* and Gram-positive *Staphylococcus aureus*. The membranes were functionalized for comparison with the antibiotic Ciprofloxacin (CIP) as a reference.

## 2. Materials and Methods

### 2.1. Chemicals and Reagents

The following chemicals were used: ethanol (EtOH, ≥99.5%), hydrochloric acid (HCl, 37.0%), malt extract, meat extract, meat peptone, potassium dihydrogen phosphate (KH_2_PO_4_), potassium iodide (KI), potato dextrose agar, potato dextrose broth, sodium chloride (NaCl), sodium dihydrogen phosphate monohydrate (NaH_2_PO_4_·H_2_O), sodium hydrogen phosphate (Na_2_HPO_4_), yeast extract, were purchased from Merck, Darmstadt, Germany. Tryptic soy broth was obtained from Fluka, Buchs, Switzerland. Calcium chloride dihydrate (CaCl_2_·2H_2_O), D-(+)-glucose anhydrous, magnesium sulfate heptahydrate (MgSO_4_·7H_2_O), manganese sulfate monohydrate (MnSO_4_·H_2_O), iron (II) sulfate heptahydrate (FeSO_4_·7H_2_O), and zinc sulphate heptahydrate (ZnSO_4_·7H_2_O) were purchased from Kemika, Zagreb, Croatia. Agar, ammonium chloride (NH_4_Cl), yeast extract, peptone from soybean, sodium carbonate (Na_2_CO_3_, ≥99.5%), and sodium hydroxide (NaOH, ≥95.0%) were purchased from Sigma-Aldrich, St. Louis, MO, USA. Ciprofloxacin (CIP, 400 mg/200 mL) was obtained from the University Medical Centre Maribor, Maribor, Slovenia.

### 2.2. Microorganisms

The selected bacterial species *E. coli* (DSM 498) and *S. aureus* (DSM 346) were purchased from DSMZ-German Collection of Microorganisms and Cell Cultures GmbH from Berlin, Germany.

### 2.3. Preparation of Mango Peel Extract

Mango peels (*Mangifera indica* L., Keitt variety) were air-dried and then extracted via ultrasound-assisted extraction (UAE), as described in our previous study [27].

### 2.4. Production of Micellar Membranes

The micellar membranes were obtained via the submerged cultivation of medicinal mushrooms. A prepared culture of the fungi *G. lucidum* or *P. ostreatus* was transferred to a previously sterilized liquid growth medium in a 250 mL Erlenmeyer flask and incubated at 27 °C under static conditions. The mycelia of both fungi were cultivated in five different growth media, the compositions of which are listed in Table 1, and at three different pH values (5.5, 7.0, 8.5) with different incubation times (2–4 weeks). The compositions and concentrations of the growth media were based on several standard fungal cultivation recipes and adjusted through preliminary experiments. The variations were designed to provide different nutrient conditions (carbon-rich, nitrogen-rich, or mineral-enriched) in order to examine their effects on the growth of micellar membranes and their properties.

After the growth of the mycelium on the surface of the liquid growth medium in the form of a membrane, this was removed from the liquid medium and purified through repeated rinsing with deionized water. Half of the micellar membranes obtained were autoclaved at 120 °C for 15 min to prevent further growth and freeze-dried. The other half of the micellar membranes were freeze-dried without pretreatment. In the further course of the study, they were sterilized via irradiation with UV light for 120 min.

### 2.5. Water-Uptake Capacity of Micellar Membranes

To determine the swelling or water-uptake capacity of micellar membranes, they were cut into pieces with a diameter of 9 mm. The dry membranes were first weighed and then immersed in deionized water at room temperature. The immersed membranes were removed from the water at certain intervals and soaked with blotting paper before being weighed again. In this way, the excess, non-adsorbed water molecules on the surfaces of the membranes were removed. The percentage of swelling was calculated using Equation (1):

Swelling (%) = ((*m*_wet,t_ − *m*_dry_)/*m*_dry_) × 100% (1)

where

*m*_wet,t_: mass of the wet micellar membrane at time t (mg),

*m*_dry_: mass of dry micellar membrane (mg).

### 2.6. Determination of Hydrophilicity/Hydrophobicity of the Surfaces of Micellar Membranes

The hydrophilicity or hydrophobicity of the micellar membrane surfaces was determined by measuring the contact angle with a Basler Aca1300–200 um digital camera with a CCTV lens (Tamron, Saitama, Japan) connected to a computer using the Open Drop algorithm at room temperature. The membrane sample was illuminated, and after adding a drop of water, the absorption of water in the membrane was recorded using a camera. The average contact angle of the lower and upper surfaces of the membrane was determined with the ImageJ program (version 1.54d, National Institutes of Health, Bethesda, MD, USA) after 5 s of contact of the water droplet with the membrane surface.

### 2.7. Chemical Characterization of Micellar Membranes

Fourier-transform infrared spectroscopy (FTIR) analysis was used to determine the presence of chemical functionalities on the surface of the obtained micellar membranes and the possible chemical interactions between MPE (or CIP) and micellar membranes in a further investigation. The spectra were obtained in the 400 to 4000 cm^−1^ range and recorded using an FTIR spectrophotometer (Shimadzu IRAffinity-1S FTIR spectrometer, Kyoto, Japan).

### 2.8. Morphological Characterization of Micellar Membranes

The morphological characterization was performed using scanning electron microscopy analysis (SEM) (FE, SEM SIRION, 400 NC and FEI) to investigate the structure of freeze-dried micellar membranes. The diameter of the hyphae and the diameters of the pores in the membranes were determined. Before analysis, the membranes were coated with a gold layer.

### 2.9. Thermal Characterization of Micellar Membranes

The micellar membranes were subjected to a thermal stability test using thermogravimetric analysis/differential scanning calorimetry (TGA/DSC) analysis. Both analyses were performed simultaneously on a TGA/DSC instrument (TGA/DSC1, Mettler Toledo AG (MTANA), Zurich, Switzerland) at a nitrogen flow rate of 50 mL/min. The samples were weighed in aluminum pans and analyzed in a 25 to 500 °C temperature range at a heating rate of 10 °C/min.

### 2.10. Functionalization of Micellar Membranes

Micellar membranes were functionalized with MPE (or with CIP as a reference) using the adsorption method. The membranes with a diameter of 9 mm were immersed in the MPE solution of different concentrations or CIP solution (2 mg/mL) at room temperature. The change in the MPE concentration or CIP was determined using a UV-VIS spectrophotometer(Varian Cary Probe 50, Agilent Technologies, Santa Clara, CA, USA). The percentage of each bioactive substance incorporated into the micellar membranes was calculated as the IE (%) using Equation (2) and as the LC (%) using Equation (3):

IE (%) = (*m*_incorporated bioactive substance_/*m*_bioactive substance_) × 100% (2)

LC (%) = (*m*_incorporated bioactive substance_/*m*_membrane_) × 100% (3)

where

IE: incorporation efficiency of the bioactive substance (MPE or CIP) into the micellar membrane (%),

LC: loading capacity of the bioactive substance (MPE or CIP) into the micellar membrane (%),

*m*_incorporated bioactive substance_: amount of bioactive substance (MPE or CIP) incorporated into the micellar membrane (mg),

*m*_bioactive substance_: amount of bioactive substance (MPE or CIP) in the initial solution (mg),

*m*_membrane_: mass of dry micellar membrane with incorporated bioactive substance (MPE or CIP) (mg).

### 2.11. In Vitro Release of Bioactive Substances from Functionalized Micellar Membranes

An in vitro study was conducted on the release of bioactive substances (MPE or CIP) from functionalized micellar membranes at body temperature (37 °C). Functionalized micellar membranes with a diameter of 9 mm were immersed in 50 mL of PBS buffer with a pH of 7.4. This was followed by incubation at 37 °C with constant shaking at 100 rpm. The samples were analyzed spectrophotometrically. The cumulative release (CR, %) of MPE from micellar membranes was calculated using Equation (4):

CR (%) = (*m*_bioactive substance,t_/*m*_incorporated bioactive substance_) × 100% (4)

CR: cumulative release of bioactive substance (MPE or CIP) from micellar membranes (%),

*m*_bioactive substance,t_: amount of bioactive substance (MPE or CIP) released from the micellar membrane at time t (mg),

*m*_incorporated bioactive substance_: amount of bioactive substance (MPE or CIP) incorporated into the micellar membrane (mg).

The release kinetics of MPE or CIP from liposomes was assessed using various mathematical models, including the zero-order, first-order, Higuchi, and Korsmeyer–Peppas models using software (OriginPro^®^ (version 10.1.0.178, OriginLab Corporation, Northampton, MA, USA)). The zero-order model describes the constant percentage of substance released over time, while the first-order model shows a logarithmic relationship between the remaining percentage of substance and time. The Higuchi model relates the percentage of substance released to the square root of time, and the Korsmeyer–Peppas model expresses the logarithmic relationship between the percentage of substance released and time [28].

The equations used for the kinetic models are as follows (Equations (5)–(8)):

*Q* = *k*_0_ × *t*, (5)

*Q* = *a* × (1 − *e*^−*k*_1_^^ × *t*^), (6)

*Q* = *k_H_* × *t*^0.5^, (7)

*Q* = *k_KP_* × *t^n^*, (8)

where

*Q*: release rate at time t [%],

*k*_0_: zero-order release constant [%/min],

*k*_1_: first-order release constant [min^−1^],

*k_H_*: Higuchi model release constant [%/min^1/2^],

*k_KP_*: Korsmeyer–Peppas model release constant [min^−*n*^],

*t*: time [min],

*a*: asymptotic constant [%],

*n*: relaxation exponent [/].

### 2.12. Determination of Antimicrobial Activity of Functionalized Micellar Membranes

To determine the inhibitory properties of functionalized micellar membranes on the growth of Gram-negative bacteria, *E. coli*, and Gram-positive bacteria, *S. aureus*, the modified disk diffusion method and the plate count method were used to determine the degree of bacterial reduction.

#### 2.12.1. Disc Diffusion Method

The inhibitory properties of micellar membranes functionalized with MPE or CIP (reference) on the growth of selected bacterial species (*E. coli* and *S. aureus*) were tested using a modified qualitative disk diffusion method. The inoculum of the tested bacteria in the optimal medium for growth with a concentration of 10^6^ CFU/mL was transferred and smeared on nutrient agar plates. Subsequently, control and functionalized micellar membranes with a diameter of 9 mm were placed on prepared agar plates, or pure MPE and CIP solution, respectively, and were applied to sterile cellulose disks. After 24 h of incubation at 37 °C, the diameter of the inhibition zone (mm) was measured.

#### 2.12.2. Plate Count Method

The plate count method was used to determine the degree of bacterial reduction of *E. coli* and *S. aureus*. Both bacterial cultures with a final concentration of 10^6^ CFU/mL in the culture medium were incubated for 24 h at 37 °C in the presence of control and functionalized micellar membranes with MPE or CIP with a diameter of 9 mm and pure solutions of MPE or CIP. The number of viable bacterial colonies was determined using tenfold serial dilutions and the smear technique on agar plates. For the determination of the size of the bacterial population expressed in CFU/mL, the agar plates were incubated at 37 °C for 24 h. The percentage of bacterial reduction was calculated using Equation (5):

Bacterial reduction percentage (%) = ((*CFU*_control_ − *CFU*_sample_)/*CFU*_control_) × 100% (9)

where

*CFU*_control_: the number of colony-forming units per mL of the control sample (control micellar membrane *G. lucidum* or *P. ostreatus*) (CFU/mL),

*CFU*_sample_: the number of colony-forming units per mL of tested sample (*G. lucidum* or *P. ostreatus*) micellar membranes functionalized with bioactive substance (MPE or CIP) or pure bioactive substance (MPE or CIP) (CFU/mL).

All analyses were performed in three replicates, and the results are given as average values. While formal statistical tests were not conducted, replicate measurements showed minimal variation and consistent trends, supporting the reliability of the reported observations.

## 3. Results and Discussion

### 3.1. Optimization of Growth Conditions for the Production of Micellar Membranes

Micellar membranes were produced via submerged cultivation of the medicinal mushrooms *G. lucidum* and *P. ostreatus*. To maximize mycelial growth with the aim of forming micellar membranes and their high-water-uptake capacity, the growth conditions, such as the composition and pH of the liquid medium and the cultivation time, were optimized.

#### 3.1.1. The Influence of the Growth Medium Composition on the Production of Micellar Membranes

In the initial optimization phase, the influence of the composition of the liquid growth medium at a pH of 5.5 on the production of both selected mushrooms was investigated. The pH of 5.5 was chosen based on medium 1, i.e., potato dextrose medium, which is characterized by an acidic pH in the range of 4.8 to 5.6 and is generally used for the cultivation of fungi. It is rich in simple sugars that can be easily digested by the fungal mycelium [6].

**Figure 1:**
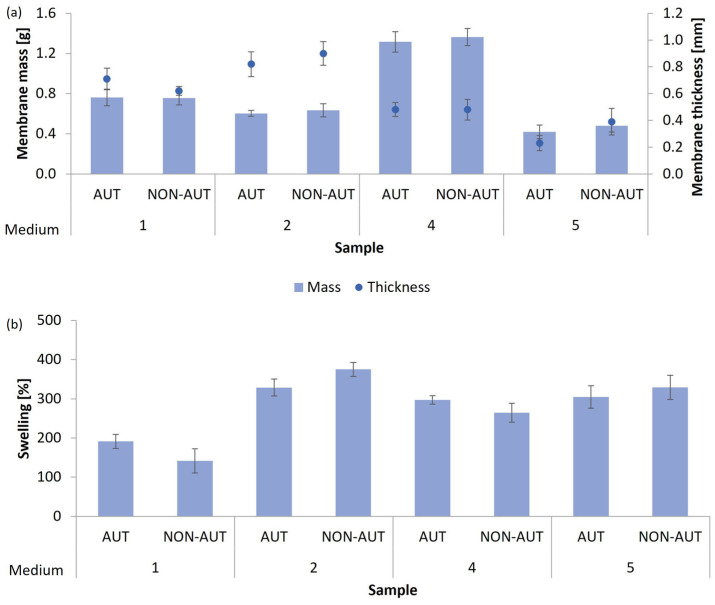
(**a**) Mass and thickness of autoclaved (AUT) and non-autoclaved (NON-AUT) micellar membranes of *G. lucidum* obtained via cultivation in different media at a pH of 5.5 and a volume of 150 mL. (**b**) Percentage of swelling in deionized water after 24 h. Media: 1—potato dextrose medium, 2—malt extract medium, 4—glucose medium, 5—medium enriched with trace elements.

**Figure 2:**
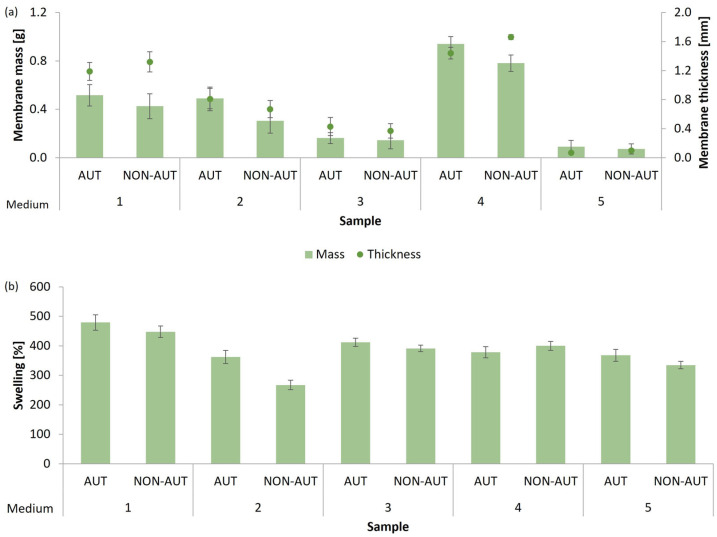
(**a**) Mass and thickness of autoclaved (AUT) and non-autoclaved (NON-AUT) micellar membranes of *P. ostreatus* obtained via cultivation in different media at a pH of 5.5 and a volume of 150 mL. (**b**) Percentage of swelling in deionized water after 24 h. Media: 1—potato dextrose medium, 2—malt extract medium, 3—peptone medium, 4—glucose medium, 5—medium enriched with trace elements.

For the production of micellar membranes, the mycelia of *G. lucidum* mushrooms or *P. ostreatus* were cultivated for 21 or 28 days in five different growth media (the composition of the growth media is listed in Table 1 in Section 2.4). The micellar membranes obtained were characterized by their mass, thickness, and ability to absorb water, expressed as the percentage of swelling after 24 h of exposure to deionized water. The results are shown in Figure 1 for *G. lucidum* and Figure 2 for *P. ostreatus*. The thickness of the micellar membranes obtained was measured using a beak scale. The results are given as mean values.

The mushroom *P. ostreatus* successfully formed micellar membranes in all five growth media tested, which were macroscopically visible as whitish floating membranes. On the other hand, *G. lucidum* did not grow in medium 3 (medium with peptone), and no micellar membranes were formed, indicating that the medium does not provide the appropriate nutrients that *G. lucidum* needs for its growth. Peptones are hydrolysates of proteins and contain mainly peptides, amino acids, and inorganic salts [29]. In addition, yeast extract was added to medium 3, a rich source of amino acids [30], that serves as a good nitrogen source. However, the absence of sugars completely prevented the growth of the *G. lucidum* mycelium.

Although the two selected mushrooms are white-rot fungi, differences in their growth and micellar membrane formation were found. The formation of micellar membranes was faster for the growth of *G. lucidum*, as it took only 14 days. With an increasing cultivation time (21 days), no further increase in growth was observed visually, which can be attributed to the consumption of the nutrients required for growth in the medium. However, the complete formation of the micellar membranes of *P. ostreatus* took 21 days. Similar to *G. lucidum*, no increase or change in membrane growth was observed with the prolongation of the incubation period to 28 days. The average mycelial growth depended on both the fungal species and the substrates or nutrients used in the growth medium. Fungal growth was also influenced by the activity of secreted enzymes for substrate degradation and nutrient uptake, which allowed the development of interwoven filamentous structures [31].

No significant differences were found between autoclaved (AUT) and non-autoclaved (NON-AUT) membranes, depending on the culture medium used. Therefore, autoclaving did not affect the possible shrinkage or reduction of membrane thickness. The highest growth of *G. lucidum* micellar membranes was obtained in medium 4—glucose medium (1.32–1.36 g, corresponding to 8.77–9.09 g membrane/L medium)—while the thickest membranes were obtained in medium 2—malt extract medium (0.82–0.90 mm). This shows that *G. lucidum* requires sugar as its preferred carbon source for optimal growth. Medium 4 provides glucose as the main sugar, whereas medium 2 contains malt extract, which is predominantly maltose. Glucose is generally preferred over other carbon compounds for fungal growth as it is rapidly metabolized to produce cellular energy [32]. The micellar membranes obtained in medium 2 also showed a remarkable ability to absorb water, expressed as the percentage of swelling (328.7–375.3%). The membranes, which were obtained in medium 4 and medium 5, enriched with traces of various elements, also absorbed a high percentage of water. However, the membranes from medium 5 were the thinnest (0.23–0.39 mm) and quite fragile. The composition of the growth medium in which the membranes are formed also influences the intensity of water uptake in the micellar membranes [31]. The ability of the films to absorb water is of great importance in biomedicine for their stability in a humid environment during application to a wound [17].

Despite the successful formation of micellar membranes of *P. ostreatus*, medium 3 and medium 5 did not allow optimal production. In medium 3, which consists mainly of peptone, the concentration of the membranes was less than 1.1 g membrane/L medium, corresponding to 0.16 g of the produced membrane, while in medium 5, the concentration of the membranes was less than 0.7 g membrane/L medium, corresponding to 0.1 g of the produced membrane. Although they could bind a high percentage of water, the membranes were very thin and fragile, making their further use difficult. It was demonstrated that the addition of trace elements had no positive effect on the growth of *P. ostreatus*. The highest increase in growth (0.78–0.94 g, corresponding to 5.21–6.27 g membrane/L medium) and the greatest thickness of micellar membranes (1.44–1.66 mm) were observed in medium 4, while the highest water uptake capacity after 24 h exposure to deionized water (448.0–479.3%) was shown by membranes produced in medium 1 (potato dextrose broth). The membranes were also characterized by the second-highest thickness achieved (1.19–1.32 mm). Similar to *G. lucidum*, *P. ostreatus* primarily requires a high proportion of sugar as a sufficient carbon source for optimal growth, as both medium 1 and medium 4 contained glucose. This is consistent with other studies confirming the successful growth of *P. ostreatus* in media with the addition of glucose as an optimal carbon source [33,34].

Based on the results obtained considering the mass and thickness of the micellar membranes, as well as the water-uptake capacity, medium 2 and medium 4 were selected for the cultivation of *G. lucidum*, and medium 1 and medium 4 were chosen for the cultivation of *P. ostreatus* for further studies. High water absorption is an important property for biomedical applications, as it helps maintain stability in humid environments during wound application and supports the binding or incorporation of extracts and other bioactive substances, both of which rely on high swelling capacity. In addition, the effects of the pH of the growth media on fungal growth and micellar membrane formation and their characteristics were additionally investigated.

#### 3.1.2. Effect of the pH Value of Growth Media on the Characteristics of Micellar Membranes

**Figure 3:**
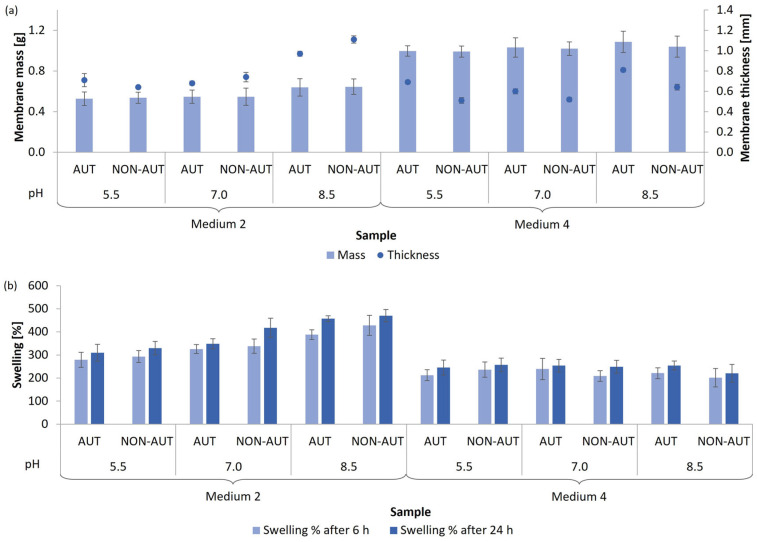
(**a**) Mass and thickness of autoclaved (AUT) and non-autoclaved (NON-AUT) micellar membranes of *G. lucidum* obtained by cultivation in medium 2 (malt extract medium) and medium 4 (glucose medium) in a volume of 150 mL after 14 days of cultivation at different pH values. (**b**) Percentage of swelling in deionized water after 6 h and 24 h.

**Figure 4:**
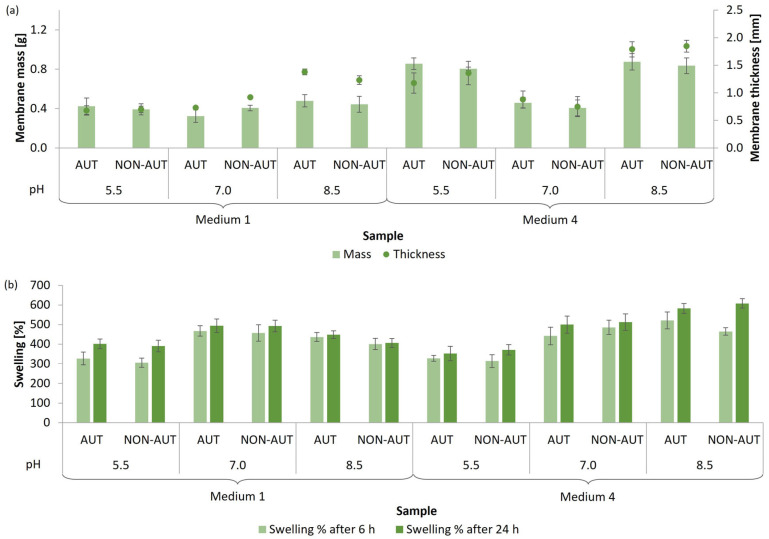
(**a**) Mass and thickness of autoclaved (AUT) and non-autoclaved (NON-AUT) micellar membranes of *P. ostreatus* obtained by cultivation in medium 1 (potato dextrose medium) and medium 4 (glucose medium) in a volume of 150 mL after 21 days of cultivation at different pH values. (**b**) Percentage of swelling in deionized water after 6 h and 24 h.

The growth of the mycelium is also strongly dependent on the pH value of the growth medium [35,36,37]. Therefore, the cultivation of both mushrooms and the subsequent production of micellar membranes was further optimized at different pH values, namely at acidic pH (5.5), neutral pH (7.0), and alkaline pH (8.5). For both fungal species, two media were selected, in which the optimal production of micellar membranes was achieved. This is directly related to the possible binding or incorporation of extracts and other bioactive substances. The results in terms of the mass and thickness of the membranes and the percentage of swelling after 6 and 24 h are shown in Figure 3 for *G. lucidum* and Figure 4 for *P. ostreatus*.

After 14 days of *G. lucidum* mycelia growth in media 2 and 4 in acidic, neutral, and alkaline environments, micellar membranes were successfully produced. Regardless of the pH value of the medium, the highest growth of micellar membranes was achieved in medium 4 (0.99–1.09 g, corresponding to 6.61–7.24 g/L medium). In general, pH had no significant effect on the mass of membranes produced with the same growth medium composition. However, the thickness of membranes produced in medium 2 at alkaline pH (0.97–1.11 mm) was slightly higher than at acidic and neutral pH. In addition, the pH value also had almost no influence on the absorption capacity of the membranes formed in medium 4. In contrast, the uptake capacity in medium 2 increased with an increasing pH and reached the highest percentage of swelling at pH 8.5 (456.8–470.0%) after 24 h.

*P. ostreatus* membranes were cultivated for 21 days. The membranes with the highest mass were formed in medium 4 with alkaline pH (0.84–0.88 g, corresponding to 5.57–5.84 g/L medium) and acidic pH (0.81–0.86 g). The thickest membranes were also produced in medium 4, having an alkaline pH. However, the pH value had no significant influence on the growth of the membranes in medium 1. Based on the calculated swelling percentages, the membranes obtained in medium 4 with alkaline pH (582.6–608.1%) and in medium 1 with neutral pH (492.8–494.6%) showed the highest water-absorption capacity after 24 h.

Overall, the percentage of swelling of the micellar membranes obtained from both mushrooms did not change significantly with an increasing exposure time from 6 to 24 h in deionized water. Furthermore, no autoclaving influence was observed on the adsorption capacity of the membranes.

Based on our results, an alkaline pH was considered optimal for the production of micellar membranes from both mushrooms. This selection was based not only on membrane growth but also on functional performance. Membranes formed under these conditions exhibited the highest water absorption, which is important for maintaining stability in humid environments and for the potential incorporation of bioactive substances in cosmetic and biomedical applications. Subedi et al. [32] investigated the mycelial growth of *G. lucidum* only in the acidic pH range. The most intensive mycelial growth was observed at pH between 4.5 and 5.5. In contrast, Ibekwe et al. [38] found that the acidic pH of 5.0 inhibited the growth of *P. ostreatus* and that optimal growth was ensured at a pH value of 6.4. When the pH increased to 7.5, there was a decrease in mycelial growth, which is consistent with our study, as the production of micellar membranes was less efficient at pH 7.0 than at pH 5.5 and 8.5. However, the different results between the studies can be attributed to the different compositions of the growth medium used. For other mushrooms, such as *Hericium erinaceus*, it has been found that a broader pH range between 5.0 and 9.0 is suitable for favorable mycelial growth [39,40]. Optimal cultivation conditions in a neutral pH environment have been confirmed for the production of micellar membranes of the fungus *Phanerochaete chrysosporium* [17].

In the following, the influence of the growth medium (medium 2 and 4 for *G. lucidum*, and medium 1 and 4 for *P. ostreatus*) and heat treatment on the hydrophilicity/hydrophobicity of the surfaces of the membranes formed and on the presence of functional groups was also investigated.

##### Effect of Growth Medium and Autoclaving of Micellar Membranes on Surface Hydrophilicity/Hydrophobicity

The further characterization of the micellar membranes concerning the influence of the growth medium, and the autoclaving of the membranes included the determination of the hydrophilicity or hydrophobicity of their surface, which is directly related to the swelling capacity or water absorption. The hydrophilicity or hydrophobicity of the surface facing upwards during cultivation (top surface) and the surface immersed in the medium during cultivation (bottom surface) of the AUT and NON-AUT micellar membranes of *G. lucidum*, obtained in media 2 and 4, and of *P. ostreatus*, obtained in media 1 and 4, were determined by measuring the contact angle of the water droplet on the membrane surface. The results for *G. lucidum* membranes are presented in Table 2, and for *P. ostreatus* membranes, they are presented in Table 3.

Depending on the interaction of a water droplet with a solid surface, the surface can be classified as hydrophilic, hydrophobic, or superhydrophobic. Surfaces with a contact angle < 90° have a strong affinity for water and are therefore defined as hydrophilic. While surfaces that show a lack of affinity for water or strongly reflect water are hydrophobic, with a contact angle > 90°. A superhydrophobic surface is defined as a surface with a contact angle greater than 150° [41].

The measured contact angles between a water droplet and the surface of *P. ostreatus* membranes, produced in both medium 1 and medium 4 were <90° regardless of the pH, indicating the hydrophilicity of both the top and bottom surfaces of each membrane according to the criterion, which is consistent with the results of very high swelling capacity from Section 3.1.2. The same applied to the bottom side of the membrane surfaces of the *G. lucidum* membranes, regardless of the pH value and the medium used. This is also directly related to the successful water-binding capacity, as the membranes were in direct contact with deionized water on their bottom side during the swelling study (Section 3.1.2). The slightly lower contact angle values of the *G. lucidum* membranes from medium 2, which indicate a stronger surface affinity for water, are consistent with the higher percentage of swelling achieved, as shown in Section 3.1.2. However, except for the membranes produced in an acidic medium, regardless of the medium, the upper surfaces of the *G. lucidum* membranes proved to be hydrophobic. This is the reason for the slightly poorer water uptake of *G. lucidum* membranes compared to *P. ostreatus*, as the highest percentage of swelling (608.1%) was achieved after 24 h of exposure to deionized water with *P. ostreatus* membranes. In comparison, the highest percentage of swelling of *G. lucidum* membranes was about 140% lower.

Other studies have also reported that AUT *P. ostreatus* membranes with a contact angle of less than 90° are more prone to absorbing moisture or water. In contrast, *G. lucidum* is reported to have a hydrophobic surface with a contact angle of about 120°. However, the study does not specify which side of the membrane (top or bottom side) was examined [22]. Similarly, Antinori et al. [21] found that the surface of the mycelial membrane of *G. lucidum* is hydrophobic. Furthermore, it was found that autoclaving the membranes showed no specific correlation with the improvement or deterioration of water-uptake capacity for the micellar membranes of both mushrooms, obtained from the same culture media at the same pH.

##### Effect of Growth Medium and Autoclaving of Micellar Membranes on the Presence of Functional Groups

FTIR analysis was used to characterize the chemical nature of the self-growing micellar membranes in relation to the different growth media used (media 2 and 4 for *G. lucidum* and media 1 and 4 for *P. ostreatus*), and the influence of heat treatment (autoclaving) was also investigated.

The FTIR spectra of micellar membranes are related to the biomolecules present, e.g., polysaccharides (O-H stretching at ~3290 cm^−1^, C-O stretching at ~1070 cm^−1^, C-C stretching at ~1020 cm^−1^, and C-H bending at ~895 cm^−1^), nucleic acids (1255–1245 cm^−1^), lipids (asymmetric and symmetric CH_2_ stretching at 3000–2800 cm^−1^), proteins (amide I at 1700–1600 cm^−1^, amide II and III at 1575–1300 cm^−1^), and chitin (C-H bending at ~1375 cm^−1^) [21,31,42].

Due to the presence of a large number of functional groups on the surface, micellar membranes from medicinal mushrooms represent a promising platform for various biotechnological applications. The presence of functional groups is shown in Table S1 for *G. lucidum* membranes and in Table S2 for *P. ostreatus* membranes.

FTIR analysis confirmed the presence of hydroxyl, carboxyl, and amide groups in the micellar membranes of the two medicinal mushrooms, which is consistent with the fact that polysaccharides, lipids, and proteins are components of the cell wall of the mushrooms. The results were comparable to the FTIR analysis of the membranes of *G. lucidum* and *P. ostreatus* in the study by Antinori et al. [22]. Regardless of the choice of growth medium, FTIR analysis confirmed slight differences between the presence of functional groups in the mycelial membranes of the two fungi. Thus, the membranes of *G. lucidum* showed more intense bands associated with lipids, which are hydrophobic molecules, suggesting that the top side of the membrane was hydrophobic. In contrast, the membranes of *P. ostreatus* had more intense bands associated with polysaccharides [6], confirming the hydrophilic nature of both the top and bottom sides of the membrane.

A broad spectrum of functional groups was observed on the surfaces of the micellar membranes of both mushrooms, indicating the potential development of micellar membranes as natural bioscaffolds for various applications. Self-growing micellar membranes are suitable for tissue engineering, as cells preferentially bind to parts of polysaccharides and proteins [43], which are present in the obtained membranes according to FTIR analysis.

The nature of growth media partially influenced the characteristics of the membranes, as there were slight differences in the FTIR spectra of the obtained micellar membranes, which could be attributed to the influence of the different nutrient compositions and pH values, which directly affected the development and growth of the mycelium. Autoclaving after the production of micellar membranes, where they were exposed to a high temperature (121 °C) and pressure (2 bar), served to inactivate the mycelia. However, their chemical structure could be altered by the high temperature and pressure, as shown in Tables S1 and S2, by the absence of certain functional groups compared to the corresponding membranes that were not autoclaved. High temperature and pressure could break or reduce glycosidic bonds and reduce the mannose content of the cell wall, the main component of mannans [44]. This was demonstrated by the absence of the mannan band in the AUT *G. lucidum* membrane from medium 2 at pH 8.5 and in AUT *P. ostreatus* membranes from medium 1 at pH 5.5 and 8.5 and from medium 4 at pH 8.5. High temperature and pressure act synergistically to inactivate the biological activity of the mycelium and at the same time cause changes in its structure. High temperatures lead to the denaturation of proteins, inactivation of enzymes, and destruction of the cell membrane of the mycelium. The high pressure during autoclaving promotes uniform heat transfer throughout the mycelium structure, ensuring more efficient inactivation and preventing mycelium regrowth while causing structural damage [22,45,46].

### 3.2. Effect of Heat Treatment on the Characteristics of Micellar Membranes

Based on the results obtained, it was determined that growth medium 2, a medium containing malt and yeast extract, was optimal for the production of *G. lucidum* membranes. Jayasinghe et al. [47] also reported that an alkaline growth medium containing malt and yeast extracts was the most suitable for the optimal growth of *G. lucidum*. The optimal membrane production of *P. ostreatus* was achieved in an alkaline medium 4 containing glucose and yeast extract, as well as traces of KH_2_PO_4_ in MgSO_4_·7H_2_O. Therefore, the preferred nutrient sources were maltose for *G. lucidum* and glucose for *P. ostreatus*.

**Figure 5:**
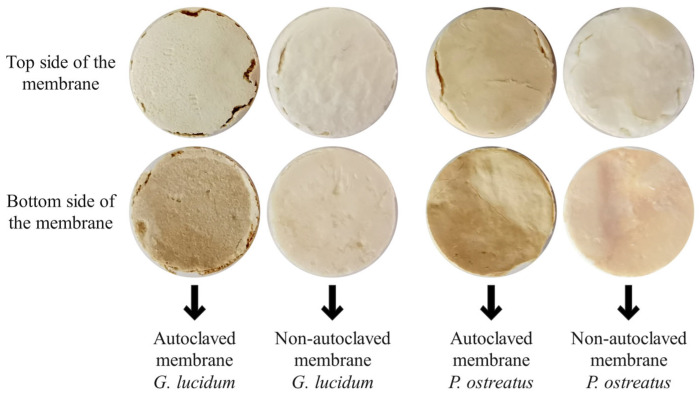
The surface facing upwards during cultivation (top surface) and the surface immersed in the medium during cultivation (bottom surface) of autoclaved and non-autoclaved micellar membranes of *G. lucidum* and *P. ostreatus*, obtained under optimal growth conditions.

In addition, the differences between AUT and NON-AUT micellar membranes have already been observed visually at the macroscopic level. AUT membranes were darker, more fragile, and showed more cracks (Figure 5). For further analysis, the effects of autoclaving on the morphological properties and the overall profile of the swelling kinetics of the micellar membranes grown in optimal media were investigated.

#### 3.2.1. Morphological Characterization of Micellar Membranes

**Figure 6:**
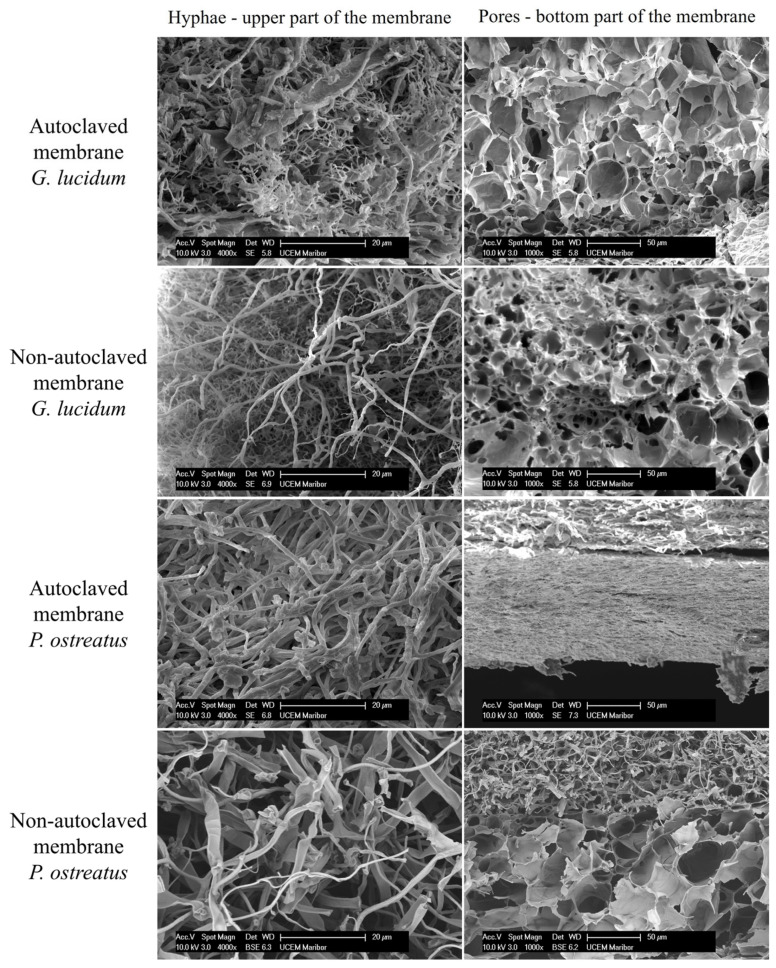
SEM images of the cross-section (upper part of the membrane—hyphae: 4000× magnification, lower part of the membrane—pores: 1000× magnification) of autoclaved and non-autoclaved micellar membranes of *G. lucidum* and *P. ostreatus*.

SEM analysis of cross-sections of AUT and NON-AUT micellar membranes of *G. lucidum* and *P. ostreatus* was used to determine the diameters and structures of hyphae and pores, which are among the most important characteristics of bioscaffolds. The results are shown in Table 4 and Figure 6.

The SEM analysis revealed that the hyphae were randomly arranged. Tubular and short hyphae, as well as long and smooth hyphae, were present in the micellar membranes. A larger average diameter of hyphae was observed in the micellar membranes of *P. ostreatus*, consistent with other studies [6,22]. On the other hand, the membranes of *G. lucidum* had a larger average pore diameter. The hyphae were located in the upper part of the membrane, while the pores were located in the lower part, which is directly related to the high-water absorption capacity, as demonstrated in the swelling study (Section 3.1.2). Since the lower side of the membrane was directly exposed to deionized water, the pores allowed water to penetrate more readily into the membrane. The *P. ostreatus* membranes showed a narrower pore band compared to the membrane of *G. lucidum*. The differences are most likely due to the two different fungal species.

AUT membranes had a denser structure because they have been exposed to a high temperature (121 °C) and pressure (2 bar). The most severe deformation was observed in the structure of the AUT membrane produced by *P. ostreatus*, indicating shrunken pores. The existing narrow band of pores was completely shrunk and deformed by the autoclaving, preventing an accurate measurement of the pore size. The fungal cell wall structure is normally shrunk or deformed when exposed to an intense heat treatment and pressure [44]. High temperature and pressure during autoclaving can cause the dehydration of the mycelium, resulting in shrinkage of the material and consequently a change in the structure and physical properties of the exposed material [48].

For a wide range of applications, including biomedical applications such as drug delivery and tissue engineering, porous materials [49] are of great importance and represent a potential platform for additional enrichment. Therefore, membranes without pores are less desirable as biocomposites for further biomedical applications.

#### 3.2.2. Swelling Kinetics of Micellar Membranes

**Figure 7:**
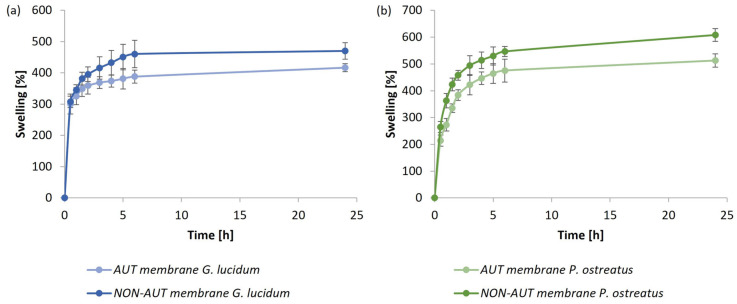
Swelling kinetics of autoclaved (AUT) and non-autoclaved (NON-AUT) membranes of *G. lucidum* (**a**) and *P. ostreatus* (**b**), obtained under optimal growth conditions.

The effect of water-uptake capacity as a function of time (up to 24 h) was studied for AUT and NON-AUT micellar membranes, obtained via cultivation in optimal media (medium 2 for *G. lucidum* and medium 4 for *P. ostreatus* at pH 8.5) that were autoclaved or not. It was investigated whether the treatment of the produced membranes influenced the overall swelling kinetic profile. The results are shown in Figure 7 for the *G. lucidum* membranes (a) and *P. ostreatus* membranes (b) as the percentage of swelling over time.

The bottom side of the micellar membranes, which was hydrophilic for both fungi according to the measured contact angles, was directly exposed to deionized water. The membranes of *G. lucidum* and *P. ostreatus* successfully absorbed water. The membranes of *G. lucidum* swelled by more than 300% after only 30 min of exposure to deionized water. With an increasing exposure time of the membrane to deionized water, there was no significant increase in the percentage of swelling (416.8% and 470.0% (after 24 h) for AUT and NON-AUT membranes, respectively). On the contrary, the *P. ostreatus* membranes absorbed the water molecules more evenly and gradually up to an exposure time of 3 h. The swelling of the membranes slowed down with an increasing exposure time. It has been found that micellar membranes form a highly interconnected network-like structure [17], which was also confirmed in our study in Section 3.2.1. Within the network system of polysaccharide fibers, a capillary force can be generated due to the porous structure, which ensures the uptake of water molecules [50,51]. The final swelling percentage of the AUT *P. ostreatus* membrane after 24 h was 512.6%. Surprisingly, the NON-AUT membrane absorbed the largest amount of water with a swelling percentage of 608.1%, more than 100% more than the NON-AUT *G. lucidum* membrane.

The differences in pore structures on the bottom side of *G. lucidum* and *P. ostreatus* membranes can be attributed to the inherent growth patterns and hyphal organization of the two fungi. Autoclaving caused partial pore shrinkage and deformation in both species, especially in *P. ostreatus*, which also influenced the water-uptake behavior.

The differences in water-absorption patterns between *G. lucidum* and *P. ostreatus* membranes can be directly related to these structural differences of their bottom-side pores observed in the SEM images. *G. lucidum* membranes have larger pores, leading to rapid initial swelling but limited further uptake over time. In contrast, *P. ostreatus* membranes exhibit smaller pores, allowing water to penetrate more gradually and evenly, resulting in higher final swelling percentages.

These observations demonstrate that both the inherent hyphal structure and the processing conditions affect the functional performance of the micellar membranes. This highlights the importance of selecting the appropriate fungal species and processing methods to tailor the functional properties of micellar membranes for biomedical applications.

Additionally, the swelling data of the micellar membranes were fitted to several kinetic models, including zero-order, first-order, Higuchi, and Korsmeyer–Peppas. For AUT and NON-AUT *G. lucidum* membranes, the release curves were best described by the Korsmeyer–Peppas model with R^2^ values of 0.941 and 0.843, respectively. The release exponent (*n* < 0.5) indicates that water uptake is governed primarily by Fickian diffusion. In contrast, AUT and NON-AUT *P. ostreatus* membranes were better described by the first-order model, with R^2^ values of 0.947 and 0.907, respectively, indicating that the rate of water uptake depends on the difference between the current and equilibrium swelling. These results highlight that the structural differences between the two fungal membranes influence both the mechanism and rate of swelling.

The produced micellar membranes are promising biomaterials when used for the development of wound healing patches as they successfully fulfill the essential criteria of patches (ensuring the reduction of water loss from the system to improve the moist environment in the wound area and protecting the wound area by limiting the loss of body fluid, as well as high mechanical strength and compatibility with the tissue system) [17,52,53]. However, the mechanical strength of the micellar membranes was not assessed in this study. Future research should evaluate their tensile properties and durability to confirm their suitability for cosmetic and biomedical applications.

#### 3.2.3. Inactivation of Mycelium

For the possible further use of micellar membranes in various applications, complete inactivation of the biological activity of the mycelium is required to stop its growth, which is usually achieved through heat treatment [20]. Antinori et al. [22] exposed the mycelial membranes to oven drying at 50 °C for 15 h or autoclaving at 120 °C for 20 min. After renewed contact with the nutrients in the potato dextrose agar (PDA), the oven-dried *G. lucidum* mycelia regrowth was observed. The drying treatment of the membranes in the oven thus proved to be an inadequate and unsuitable method. On the other hand, the oven-dried mycelium of *P. ostreatus* was inactivated entirely, as was the autoclaved mycelium of both mushrooms. The heat resistance of *G. lucidum* strains in contrast to *P. ostreatus* has also been reported by Zhang et al. [45].

**Figure 8:**
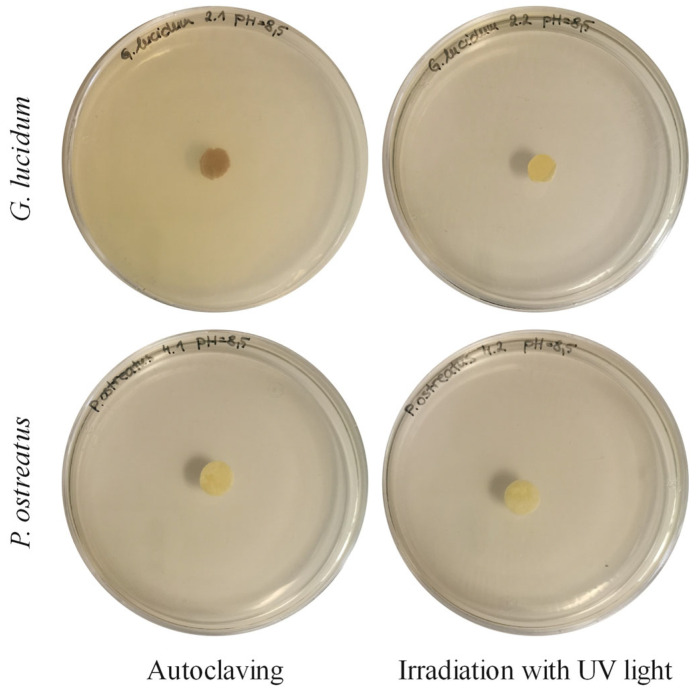
Autoclaved and UV-irradiated membranes of *G. lucidum* and *P. ostreatus* after 30 days of incubation at 27 °C on PDA plates.

The results of our study have shown that autoclaving caused changes in the structure and absorption capacity of water molecules. Therefore, the NON-AUT membranes were sterilized in a laminar chamber under UV light for 2 h to prevent further mycelium growth. A study was carried out on the effects of irradiation with UV light on the inactivation of the mycelium compared to AUT micellar membranes. The previously irradiated and swollen micellar membranes of both mushrooms were applied to PDA plates, and possible growth was observed. After 30 days (Figure 8), no regrowth of the mycelium was observed in any of the cases, indicating successful inactivation, even when the membranes were only exposed to UV light. This is a very favorable result for further studies and possible biomedical applications. The fungicidal effect of irradiation with UV light (254 nm) has been confirmed in various studies [54,55]. Irradiation with UV light is considered an environmentally friendly approach with sufficient fungicidal activity, including inactivation of the spores and minimal impact on the treated object [56]. For this reason, the NON-AUT micellar membranes of both mushrooms were selected for further studies on membrane functionalization with MPE or CIP and their characterization.

### 3.3. Functionalization of Micellar Membranes

**Figure 9:**
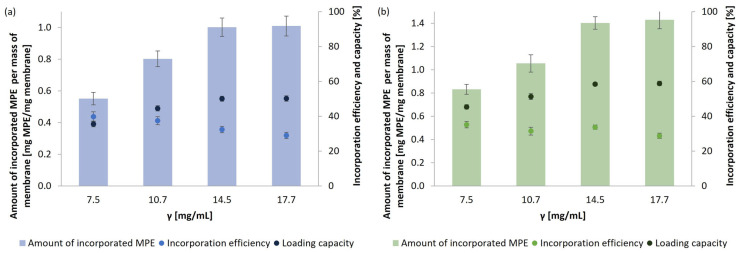
Incorporation efficiency (IE), loading capacity (LC), and amount of incorporated MPE per mass of micellar membranes of *G. lucidum* (**a**) and *P. ostreatus* (**b**) at different concentrations of MPE.

**Figure 10:**
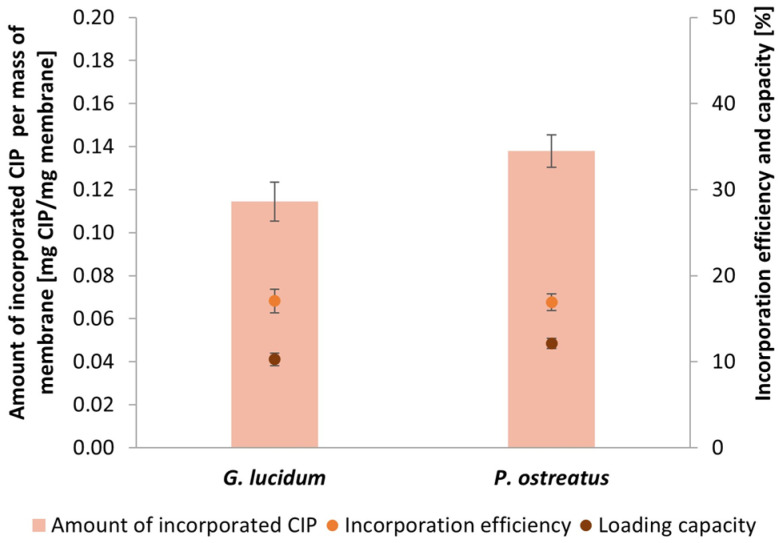
Incorporation efficiency (IE), loading capacity (LC), and amount of incorporated CIP (2 mg/mL) per mass of micellar membranes of *G. lucidum* and *P. ostreatus*.

There is a strong trend toward developing and using biocompatible, biodegradable, and non-toxic scaffolds for various biomedical applications. Self-grown micellar membranes from medicinal mushrooms are a promising candidate for potential use in tissue engineering and wound healing. In order to increase their potential for use in biomedical applications, the membranes were functionalized through adsorption with MPE. MPE is an excellent source of various bioactive substances with antioxidant and antimicrobial properties, as already found in our previous study [27]. Different concentrations of MPE were incorporated into the micellar membranes (63.6 mm^2^) to achieve the highest possible LC of MPE in the membranes from both mushrooms. The results are shown in Figure 9a for *G. lucidum* and Figure 9b for *P. ostreatus*. As a reference, the membranes were functionalized with the antibiotic CIP at a concentration of 2 mg/mL (Figure 10).

MPE has been successfully incorporated into fibrous micellar membranes, confirming the possibility of membrane functionalization and the resulting improvement in the biological activity of micellar membranes for potential use in various biomedical applications. The amounts of incorporated MPE per mass of membranes and IE were comparable in the micellar membranes obtained from both selected medicinal mushrooms. The amount of incorporated MPE per mass of membranes increased with an increasing initial MPE concentration, and consequently, IE decreased as it depended on the amount of MPE in the initial solution. The LC, calculated concerning the dry mass of the functionalized membranes, increased with an increasing MPE concentration until the maximum possible LC of MPE in the membranes was reached, i.e., 50.05% and 58.37% in the membranes of *G. lucidum* and *P. ostreatus*, respectively. The slightly higher LC in the *P. ostreatus* membrane was due to a higher absorption capacity compared to the *G. lucidum* membrane, as presented in Section 3.2.2. As noted, IE strongly depended on the origin of the membrane, which was confirmed in other studies. Khamrai et al. [17] achieved 93% IE of the pure bioactive substance curcumin in micellar membranes of *P. chrysosporium*.

CIP (2 mg/mL) was incorporated into the membranes as a reference (Figure 10). As expected, it was found that comparable IE and LC were achieved in the membranes from both fungal species. Antibiotic molecules are generally small molecules compared to molecules of polyphenolic compounds in MPE, which, in the case of CIP, resulted in a much poorer IE, as it was much more challenging to capture the small CIP molecules in the porous and fibrous structure of the micellar membranes. The LC percentage of CIP was only 10.27% and 12.12% in *G. lucidum* and *P. ostreatus* membranes, respectively. These results indicate that micellar membranes are suitable for the incorporation of larger molecules, such as various bioactive substances and natural extracts.

### 3.4. In Vitro Release of Bioactive Substances from Functionalized Micellar Membranes

**Figure 11:**
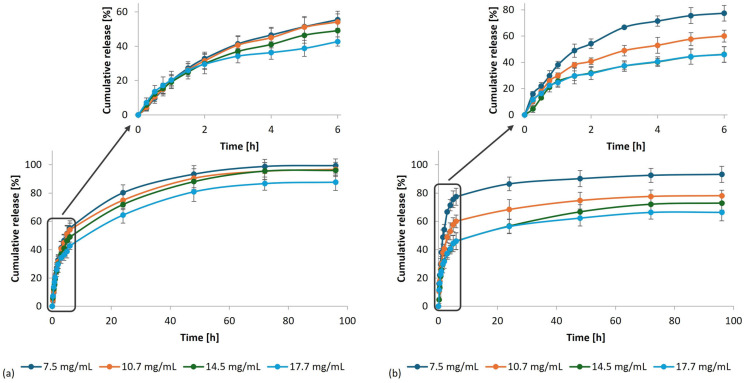
Release kinetics of MPE from micellar membranes of *G. lucidum* (**a**) and *P. ostreatus* (**b**) as a function of different MPE concentrations in PBS, pH 7.4, at 37 °C and 100 rpm in 96 h.

The in vitro release of the bioactive substances from the micellar membranes was also investigated at 37 °C in PBS with shaking at 100 rpm. The release kinetics of MPE at different concentrations from the membranes are presented in Figure 11 (*G. lucidum* (a) and *P. ostreatus* (b)).

The rapid release of MPE from *P. ostreatus* membranes at all MPE-loaded concentrations was observed during the first 5 h of the release study. After 24 h, the release of MPE from *P. ostreatus* stabilized completely. In contrast, MPE was released uniformly and gradually from *G. lucidum* membranes regardless of the MPE-loaded concentration. Despite the rapid release of MPE from the *P. ostreatus* membranes in the initial phase, except from the membrane with the lowest incorporated MPE concentration (93.25%), no more than 80% of the MPE was released. At the highest concentration, however, the percentage of MPE released did not even exceed 70%.

**Figure 12:**
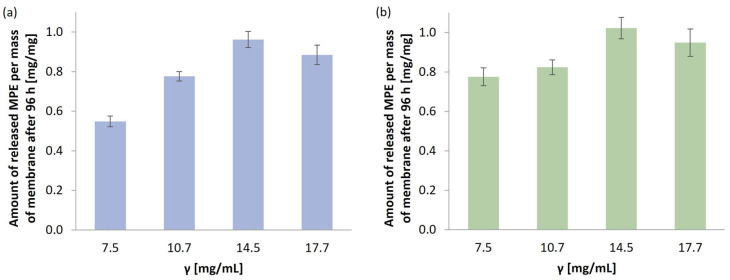
Total amount of released MPE per mass of membrane after 96 h from *G. lucidum* (**a**) and *P. ostreatus* (**b**) micellar membranes as a function of the MPE concentration loaded into the membrane.

On the other hand, MPE was almost entirely released from the *G. lucidum* membranes, with the final percentage ranging from 87.67% to 99.46% (between the lowest and highest MPE concentrations studied). The reason could be due to the differences in the *P. ostreatus* membrane structure and consequently stronger MPE incorporation. As the SEM analysis showed, the *P. ostreatus* membrane contained only a narrow band of pores, while most of the membrane consisted of hyphae forming a fibrous structure between which the MPE could be more strongly embedded. Therefore, it could not be completely released from the membranes at pH 7.4 and 37 °C in PBS. This is also reflected in the total amount of released MPE per mass of membranes after 96 h of release (Figure 12).

The total concentration of MPE released in the in vitro release study (after 96 h) in 50 mL of PBS, pH 7.4, at 37 °C increased with an increasing MPE concentration up to a concentration of 14.5 mg/mL and then slightly decreased. It was found that the highest amount of MPE released was 0.96 mg per mass of *G. lucidum* membrane and 1.02 mg/mg per mass of *P. ostreatus* membrane. Based on these results, further characterizations (SEM, FTIR, and TGA/DSC) were performed for the membranes with incorporated MPE at a concentration of 14.5 mg/mL, and the antimicrobial potentials of such membranes were verified.

Khamrai et al. [17] also achieved the gradual release of curcumin from the *P. chrysosporium* mycelial membrane with a maximum release percentage of 90% after 96 h. The therapeutic efficacy of a transdermal wound healing system depends primarily on the kinetics of the incorporated bioactive substances’ release from the carrier system. The gradual and sustained release of the embedded bioactive substance from the biocomposites enables increased availability and precise dosing of the bioactive substance at the treated site, improves the stability of the bioactive substance, and provides protection against too rapid degradation [17,57,58].

**Figure 13:**
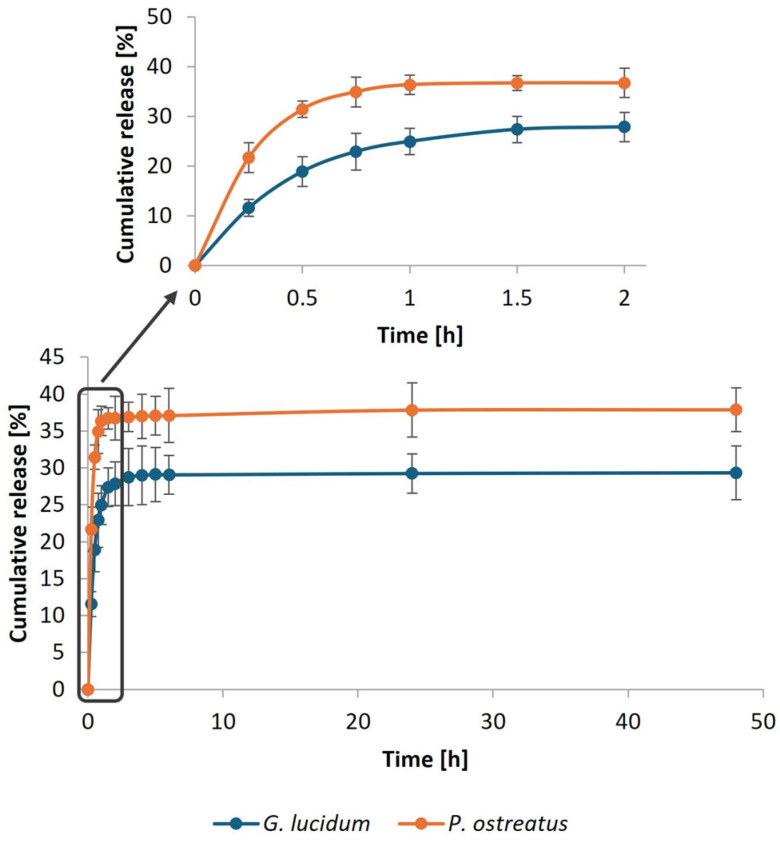
Release kinetics of CIP (2 mg/mL) from *G. lucidum* and *P. ostreatus* micellar membranes in PBS, pH 7.4, at 37 °C and 100 rpm for 48 h.

Similar to the incorporation of MPE into micellar membranes, faster release of CIP was observed from *P. ostreatus* membranes than from *G. lucidum* membranes (Figure 13). However, the overall release profile of CIP was quite different. The uniform release of CIP from both membranes was achieved for up to 1 h, after which the release began to slow down. After 48 h, 29.35% of the CIP (0.03 mg CIP/mg membrane) was released from *G. lucidum* and 37.90% (0.05 mg CIP/mg membrane) was released from *P. ostreatus* membranes. The reason for the low percentage of CIP released was the stronger incorporation of CIP into micellar membranes due to the possible formation of interactions between CIP and micellar membranes. CIP adsorbed on poly(lactic-co-glycolic) acid (PLGA) disks was also not completely released from the polymer structures, indicating the formation of strong interactions between CIP and PLGA [59]. Baskakova et al. [60] also reported that the release of CIP from electrospun poly(ε-caprolactone) (PCL) fibers was limited precisely because of the strong interactions between CIP and PCL.

To better understand the behavior and mechanism of bioactive substance release from the *G. lucidum* and *P. ostreatus* micellar membranes, the experimental data from the in vitro release study were fitted to four commonly used kinetic models (zero-order, first-order, Higuchi, and Korsmeyer–Peppas models). Experimental data obtained for the MPE release at a concentration of 14.5 mg/mL were used to determine the optimal model describing the release of MPE from membranes. The best fit to the experimental data and, thus, the most appropriate kinetic model was determined using the correlation coefficient (R^2^). The determined kinetic parameters and R^2^ are presented in Table 5.

Among all four kinetic models used to analyze the best fit of the bioactive substance release profile from the micellar membranes, it was found that for the release of MPE (14.5 mg/mL) from the both mushroom micellar membranes, the best correlation was with the Korsmeyer–Peppas model, as the R^2^ value was closest to 1.

**Figure 14:**
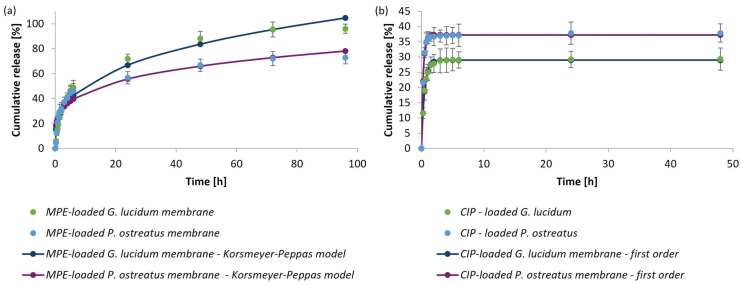
Experimental results fitted to the theoretically predicted release of MPE (**a**) and CIP (**b**) from *G. lucidum* and *P. ostreatus* micellar membranes.

Figure 14a further presents the experimental results fitted to the theoretically predicted MPE release from both mushroom micellar membranes according to the Korsmeyer–Peppas model. The Korsmeyer–Peppas model describes the release of a bioactive substance from polymeric carriers and suggests diffusion-controlled release. The model provides an exponential correlation between the bioactive substances release from the carrier and time [61]. It is useful when the mechanism of release is unknown or when more than one type of release phenomenon is involved [62]. The diffusion exponent (parameter *n*) can be used to determine the release mechanism [63]. If *n* < 0.45, the release corresponds to Fickian diffusion; if 0.45 < *n* < 0.89, it corresponds to non-Fickian diffusion or irregular diffusion; if *n* = 0.89, the release mechanism represents case II transport; and if *n* > 0.89, it indicates super case II transport for cylindrical geometries [64,65]. The experimentally determined values of *n* were below 0.45 for all samples, which corresponds to a Fickian diffusion mechanism in which the transport of molecules is driven by a concentration gradient. In contrast, a first-order model can adequately describe the release of CIP (2 mg/mL) from both mushrooms micellar membranes, as the R^2^ value was 0.999 in both cases. Figure 14b presents the experimental results fitted to the theoretically predicted CIP release from both mushroom micellar membranes according to the first-order release mechanism. The first-order model describes the concentration-dependent release of the bioactive substance from different systems.

### 3.5. Characterization of Functionalized Micellar Membranes

The membranes from *G. lucidum* and *P. ostreatus* functionalized with MPE (14.5 mg/mL) and CIP (2 mg/mL) were further characterized via FTIR, SEM, and TGA/DSC analyses to determine the chemical, morphological, and thermal properties of the functionalized membranes. The MPE concentration of 14.5 mg/mL was chosen based on the results of the previous section, where in this case, the highest LC and the highest amount of MPE released from both mycelial membranes were observed.

#### 3.5.1. Morphological Characterization of Functionalized Micellar Membranes

**Figure 15:**
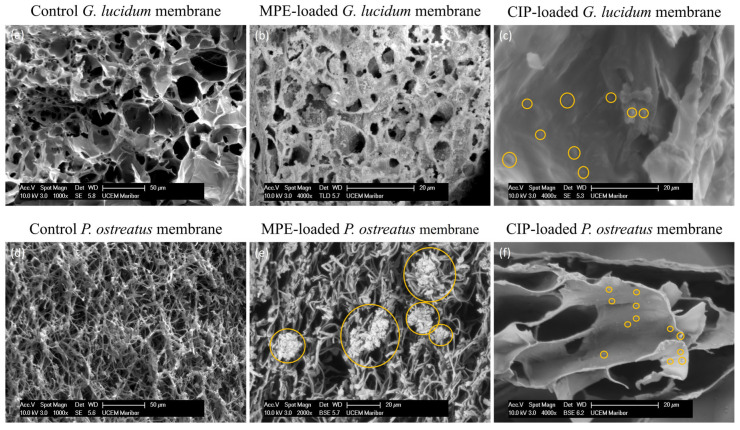
SEM images of control micellar membranes from *G. lucidum* (**a**) and *P. ostreatus* (**d**) and of MPE- (**b**,**e**) and CIP-functionalized (**c**,**f**) membranes at different magnifications.

The morphological characterization of the functionalized micellar membranes from *G. lucidum* and *P. ostreatus* with MPE (14.5 mg/mL) and CIP (2 mg/mL), respectively, was performed via SEM analysis. The obtained SEM images are shown in Figure 15. A detailed morphological characterization of the micellar control membranes from both mushrooms has already been described in Section 3.2.1.

MPE (14.5 mg/mL) was uniformly incorporated into the entire porous structure of the micellar membrane of *G. lucidum*. This was the reason for the gradual and uniform release of MPE from the membrane (>90%), as observed in the in vitro release study in Section 3.4. The control membranes exhibited a smoother surface morphology (Figure 15a), while the morphology of the functionalized membranes was rougher (Figure 15b), confirming the successful incorporation of MPE into the mycelial matrix. The measured pore diameters further confirmed the incorporation of MPE into the micellar membrane. The control membranes had a diameter of 40.30 ± 9.83 µm, while the average pore size of the MPE-functionalized membranes was 4.29 ± 1.47 µm. A similar reduction of pores via the adsorption of proteins to biomimetic chitosan/hydroxyapatite scaffolds was found in a study by Nga et al. [66], confirming the successful functionalization of the scaffold. Due to the narrow pore band in the membrane of *P. ostreatus* compared to *G. lucidum*, the MPE was mainly embedded or enclosed in the fibrous structure of the *P. ostreatus* membrane in the form of aggregates and less in the pores themselves (Figure 15e). For this reason, the release from *P. ostreatus* membranes was faster in the initial phase. However, it is possible that some of the polyphenolic molecules in the MPE were more tightly intertwined in the hyphae, so the final percentage of MPE released could not exceed 80%. Figure 15c (*G. lucidum* membrane with CIP) and Figure 15f (*P. ostreatus* membrane with CIP) also show the successful incorporation of CIP molecules into micellar membranes (highlighted with orange circles).

#### 3.5.2. Thermal Stability of Functionalized Micellar Membranes

**Figure 16:**
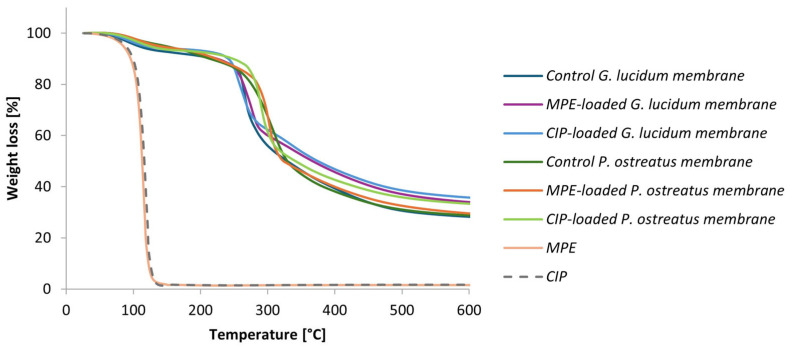
TGA thermograms of control and with MPE- or CIP-functionalized micellar membranes of *G. lucidum* and *P. ostreatus*, as well as of free MPE and CIP.

Thermal stability of the control and the functionalized micellar membranes, as well as of the free MPE or CIP, was tested via TGA/DSC analysis. The thermal degradation of the samples determined via the TGA analysis is shown in Figure 16 as weight loss over the temperature range of 25–600 °C. Table 6 presents the total weight loss of control and with MPE or CIP functionalized *G. lucidum* and *P. ostreatus* micellar membranes.

A drastic weight loss of free MPE and CIP was observed from 60 to 165 °C. The percentage weight loss was more than 98% in both cases. Biphasic weight loss was observed in the control membranes and the functionalized micellar membranes of both mushrooms. The first phase of weight loss of the *G. lucidum* membranes occurred between 42 and 209 °C and resulted in a weight loss of 6.3–10.0%. A similar thermal degradation profile was observed for the *P. ostreatus* membranes. The first phase of weight loss is related to water evaporation and the degradation of low-molecular-weight amphiphilic proteins, the hydrophobins [67]. The next phase of weight loss resulted in a weight loss of 61.2% (212–600 °C), 58.0% (187–602 °C), and 57.1% (174–594 °C) for the control and MPE- and CIP-functionalized G. *lucidum* membranes, respectively. A weight loss of 63.1% (179–595 °C), 62.7% (180–599 °C), and 58.5% (188–599 °C) was observed in the second phase for the control and MPE- and CIP-functionalized *P. ostreatus* membranes, respectively. The second phase of weight loss is due to the thermal degradation of the cell wall polysaccharides and proteins present in the hyphal scaffold [20]. The results of the micellar control membranes are in agreement with the reviewed literature, which also reports a biphasic weight loss profile [6,20,31].

The high degradation temperature of both control and functionalized micellar membranes shows that the micellar membranes of *G. lucidum* and *P. ostreatus* are stable, which offers excellent potential for their application in a broader range of fields [6].

**Figure 17:**
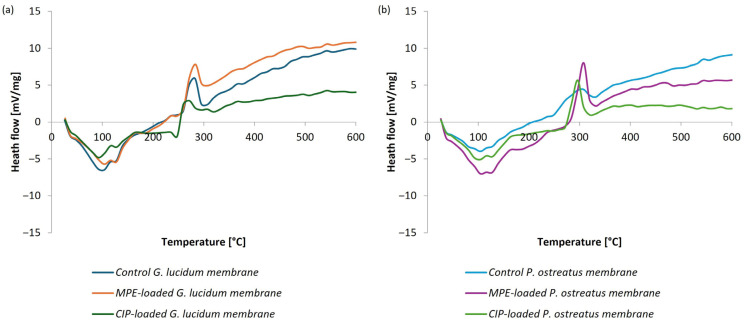
DSC thermograms of control and functionalized micellar membranes of (**a**) *G. lucidum* and (**b**) *P. ostreatus* with MPE or CIP.

The phase behavior of the samples was further investigated via DSC analysis in a temperature range from 25 to 600 °C. The DSC thermograms of the control and the functionalized micellar membranes are shown in Figure 17 ((a) for *G. lucidum* and (b) for *P. ostreatus*).

DSC thermogram of the free MPE and CIP showed a sharp endothermic melting peak at 120 °C and 123 °C, respectively. In contrast, the DSC profile of the control micellar membranes from both *G. lucidum* and *P. ostreatus* showed a weak endothermic peak in the temperature range of 90 °C to 110 °C, which was attributed to water evaporation. In the loaded membranes, the melting peaks of CIP and MPE disappear or become markedly reduced. This suggests that the compounds are well dispersed within the polysaccharide matrix and may interact with it, for example through hydrogen bonding, which reduces their crystallinity. The characteristic exothermic peaks of the micellar membranes occur in the temperature range around 250 °C and 320 °C, corresponding to the degradation of the micellar matrix. Their onsets and peak temperatures are similar or only slightly shifted relative to the unloaded control membranes, indicating that loading does not compromise matrix thermal stability and stabilizes the incorporated compounds against melting or recrystallization.

These findings provide valuable insights into the stability of the controlled and functionalized micellar membranes, confirming their storage and potential further processing without significant thermal degradation.

Overall, the DSC results are in agreement with the TGA findings and confirm that both control and loaded micellar membranes obtained from both medicinal mushrooms are thermally stable within the studied temperature range. The thermal stability of MPE and CIP is also improved when they are incorporated into a micellar matrix.

#### 3.5.3. Chemical Characterization of Functionalized Micellar Membranes

**Figure 18:**
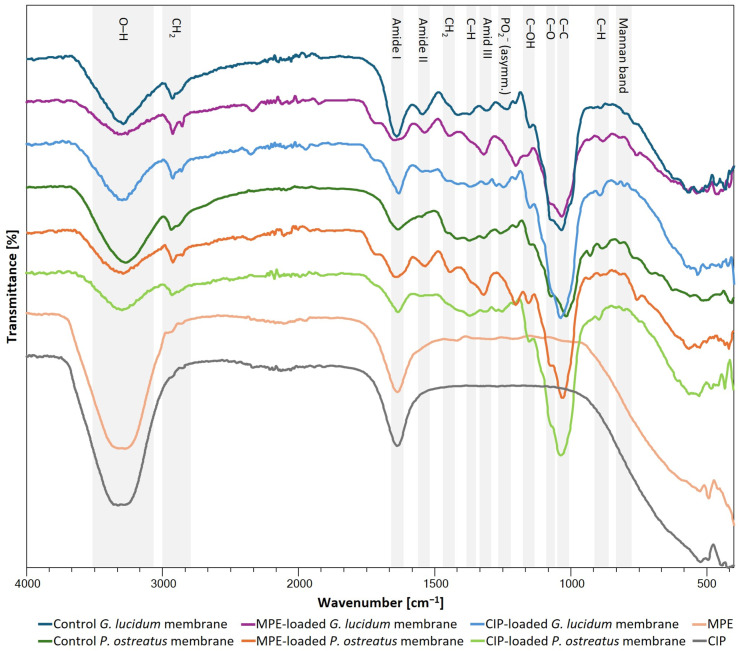
FTIR spectra of control and functionalized micellar membranes from *G. lucidum* and *P. ostreatus* with MPE or CIP, as well as free MPE and CIP.

FTIR analysis was performed to determine the possible occurrence of molecular interactions between the mycelial matrix and the embedded MPE or CIP. The obtained FTIR spectra in the range of 400 to 4000 cm^−1^ are shown in Figure 18.

The FTIR spectrum of the MPE solution shows a characteristic peak at 3271 cm^−1^, characteristic of the O-H group. The peaks at 2947 cm^−1^ and 2859 cm^−1^ are due to the asymmetric and symmetric vibrations of CH_2_, respectively, and the peak at 1636 cm^−1^ is due to vibrations of the C=C group. The peaks in the 1600–1200 cm^−1^ range are consistent with the presence of polyphenols in MPE [68]. The FTIR spectrum of CIP showed two prominent peaks at 3321 cm^−1^ and 1636 cm^−1^, corresponding to O-H group and C=C group vibrations, and peaks at 2930 cm^−1^ and 2853 cm^−1^ due to asymmetric and symmetric CH_2_ vibrations, respectively, similar to those reported in the literature [69].

In Section Effect of Growth Medium and Autoclaving of Micellar Membranes on the Presence of Functional Groups, the functional groups present in the *G. lucidum* and *P. ostreatus* micellar membranes (Tables S1 and S2, Section Effect of Growth Medium and Autoclaving of Micellar Membranes on the Presence of Functional Groups) were determined before functionalization. The FTIR spectra of the membranes obtained from the two medicinal mushrooms with the incorporated bioactive substance were similar to those of the pure micellar membranes (Figure 18). MPE- and CIP-loaded micellar membranes, namely, showed all important characteristic peaks as the corresponding control membranes, i.e., the representative groups of polysaccharides (O-H stretching, C-OH stretching, C-O stretching, C-C stretching, glucan β-anomer C-H bending, mannan band, C-H bending (chitin), lipids (CH_2_ asymmetric and symmetric stretching), and proteins (amide I and II). Thus, no significant changes in the characteristic peaks could be detected, indicating that the chemical groups of the micellar membranes have not changed significantly with the incorporation of the bioactive substances. Thus, no new chemical bonds were formed between the mycelial matrix and the incorporated bioactive substances that could alter their properties. This indicates the relative stability of MPE and CIP when incorporated into micellar membranes. Some minor differences in the absorbance peaks of the functionalized membranes compared to the pure membranes were due to the overlap of the absorbance peaks of the functional groups of the incorporated bioactive substance (MPE or CIP) with the peaks of the pure micellar membranes. Similar results, namely the absence of chemical interactions between the polymer matrix and the embedded drug (prodigiosin or paclitaxel), were obtained by Obayemi et al. [63] in the prepared porous PLGA-PEG and PLGA-PCL scaffolds. Khamrai et al. [17] also confirmed the stability of curcumin incorporated into the biomass of *P. chrysosporium* mycelia, as no significant changes in the characteristic peaks were detected via FTIR analysis.

### 3.6. Antimicrobial Properties of Functionalized Micellar Membranes

Functionalized membranes with natural extracts may significantly contribute to the therapeutic effect. They can successfully inhibit the growth and development of microorganisms due to the presence of a spectrum of bioactive substances with antimicrobial properties. A sustained-release profile can achieve sufficient antimicrobial activity over a longer period of time. The antimicrobial potential was validated by performing a modified qualitative disk diffusion method on nutrient agar plates, whereby the inhibition zones were determined after 24 h of incubation. The inhibitory effect of functionalized micellar membranes compared to control membranes and MPE solution on the growth of *E. coli* and *S. aureus* was studied. CIP-functionalized micellar membranes and CIP solution served as a positive reference. Table 7 shows the measured inhibition zone diameters (mm) after exposure of the individual bacterial species to the samples.

The *G. lucidum* and *P. ostreatus* control membranes showed no inhibitory effect on the growth of the tested bacteria, *E. coli* and *S. aureus*, as no growth inhibition zone was detected after 24 h of incubation at 37 °C, indicating that the control micellar membranes have no antimicrobial activity. In contrast, the antimicrobial properties of the functionalized micellar membranes with incorporated MPE or CIP were successfully determined based on the measured inhibition zones. This further confirms the successful incorporation of bioactive agents into the micellar membranes of both selected medicinal mushrooms and the active antimicrobial effect due to the gradual release of the incorporated substances from the membranes. For comparison, functionalized *P. chrysosporium* micellar membranes with curcumin also showed effective inhibitory properties on the growth of *E. coli*, *P. aeruginosa*, *S. aureus*, and *Lysinibacillus fusiformis* [17].

**Figure 19:**
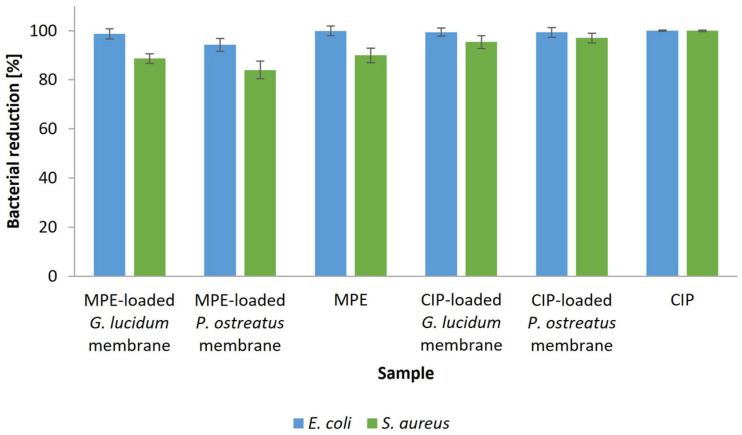
Bacterial reduction (%) after 24 h exposure of *E. coli* and *S. aureus* to control and functionalized micellar membranes with MPE or CIP and free MPE or CIP.

In addition, the antibacterial efficacy of the functionalized micellar membranes and the pure solutions of bioactive substances (MPE and CIP, respectively) was tested using a more accurate and reliable plate count method, and the percentage of bacterial reduction after a 24 h exposure to the samples was determined. The results are shown in Figure 19.

Successful growth inhibition was achieved after both tested bacteria were exposed to the functionalized membranes. When testing the inhibitory properties of both bacteria, a slightly higher reduction rate of 98.7% and 88.6% was achieved for *E. coli* and *S. aureus* after exposure to the MPE-functionalized *G. lucidum* membrane than with the MPE-functionalized *P. ostreatus* membrane (94.2% and 84.0%, respectively). This is consistent with the in vitro release study (Section 3.4), as more MPE was released from the membrane of *G. lucidum* (72.0%) than from that of *P. ostreatus* (56.7%) after 24 h. Since the amount of MPE released in the in vitro release study increased with an increasing exposure time in PBS, the functionalized micellar membranes represent promising natural biocomposites that could provide antimicrobial activity over a prolonged period of time in addition to their therapeutic effects. The same applies to CIP-functionalized membranes. The functionalized *G. lucidum* membrane achieved a partially higher bacterial reduction rate, as a higher percentage of CIP was released in an in vitro release study after 24 h of exposure to PBS. It is also important to note that the results of the plate count method are comparable to those of the disk diffusion method.

## 4. Conclusions

Micellar membranes, which could be produced from the medicinal mushrooms *G. lucidum* and *P. ostreatus*, are self-growing, purely natural materials with unique properties based primarily on their biodegradability and biocompatibility. Their spontaneously formed three-dimensional biopolymer network and fibrous structure pave the way for innovative polymeric biocomposites that can be used in various industries, including biomedical applications.

Micellar membranes of microbial origin have been investigated as a promising advanced delivery system. When optimizing the cultivation of the medicinal mushrooms *G. lucidum* and *P. ostreatus* via submerged cultivation, a significant influence of the composition and pH of the growth medium on the obtained micellar membranes and their characteristics was observed. The highest growth and, thus, the most intensive formation of micellar membranes with a high-water uptake capacity was achieved when cultivating *G. lucidum* (14 days) in malt extract medium and *P. ostreatus* (21 days) in glucose medium. Therefore, the best sources of nutrients were sugar, maltose, and glucose for *G. lucidum* and *P. ostreatus*, respectively. The most successful fungal growth is usually achieved in acidic pH growth media, whereas in our study, the most successful fungal growth and subsequent production of micellar membranes with desired characteristics was achieved in alkaline growth media.

Micellar membranes have shown a high-water absorption capacity due to their hydrophilic nature, which makes them particularly potential biomaterials for biomedical applications as they successfully fulfill the criterion of persistence in a humid environment during application to a wound. However, this requires complete inactivation of the biological activity of the mycelium, which can only be achieved by irradiating the membranes with UV light, thus ensuring complete inactivation of the mycelium without deforming the structure of the micellar membranes.

In addition, micellar membranes obtained from both therapeutic mushrooms were successfully functionalized using MPE and CIP, respectively. The LC of the bioactive substance incorporated into the membranes of *G. lucidum* and *P. ostreatus* was 50.05% and 58.37% for MPE and 10.27% and 12.12% for CIP, respectively. FTIR analysis showed the presence of a wide range of functional groups and confirmed the stability of the bioactive substance incorporated into the membranes. Also, the thermal stability of control and functionalized micellar membranes was confirmed, as was the presence of hyphae and pores in the membranes and the successful incorporation of the bioactive substance into the membranes. In addition, the in vitro release study showed a gradual and uniform release of MPE from the micellar membranes compared to CIP, of which only 40% was released from the membranes. Furthermore, membranes with incorporated bioactive substances showed antimicrobial properties with a high degree of *E. coli* and *S. aureus* growth inhibition.

Micellar membranes are innovative biocomposites that are suitable for various biomedical applications. Functionalized with diverse natural extracts such as MPE, they represent a source of bioactive substances that have many positive effects on health due to their antioxidant, antimicrobial, and many other activities. Such biocomposites represent a promising platform for tissue engineering, wound healing, and innovative skin materials as they mimic the extracellular matrix of the skin. However, further studies are needed to investigate the cytotoxicity of micellar membranes and the interactions between cells and tissues and the mycelium.

## Figures and Tables

**Table 1:** Composition of growth media for the production of micellar membranes.

Growth Medium	Composition	Concentration (g/L)
Medium 1—Potato dextrose medium	Potato dextrose broth	24.0
Medium 2—Malt extract medium	Malt extract	10.0
	Yeast extract	4.0
Medium 3—Peptone medium	Peptone	5.0
	Yeast extract	2.0
	KH_2_PO_4_	1.0
	MgSO_4_·7H_2_O	0.5
	MnSO_4_·H_2_O	0.075
Medium 4—Glucose medium	Glucose	49.2
	Yeast extract	4.9
	KH_2_PO_4_	0.88
	MgSO_4_·7H_2_O	0.5
Medium 5—Medium enriched with trace elements	Glucose	10.0
	NH_4_Cl	4.0
	Yeast extract	1.0
	CaCl_2_·2H_2_O	0.4
	KH_2_PO_4_	0.25
	MgSO_4_·7H_2_O	0.25
	FeSO_4_·7H_2_O	0.013
	ZnSO_4_·7H_2_O	0.004

**Table 2:** Hydrophilicity or hydrophobicity of the surfaces of the autoclaved (AUT) and non-autoclaved (NON-AUT) micellar membranes of *G. lucidum* obtained in 2 and 4 according to the measured contact angles.

Medium	pH	Sample	Top Side of the Membrane		Bottom Side of the Membrane	
			Average Contact Angle	Hydrophilic or Hydrophobic Surface	Average Contact Angle	Hydrophilic or Hydrophobic Surface
**Medium 2**	5.5	AUT	83.94 ± 3.55°	Hydrophilic	41.57 ± 1.95°	Hydrophilic
		NON-AUT	75.31 ± 6.32°	Hydrophilic	43.07 ± 1.34°	Hydrophilic
	7.0	AUT	97.20 ± 2.20°	Hydrophobic	33.78 ± 1.83°	Hydrophilic
		NON-AUT	134.12 ± 0.27°	Hydrophobic	35.73 ± 2.26°	Hydrophilic
	8.5	AUT	96.53 ± 1.10°	Hydrophobic	34.29 ± 2.48°	Hydrophilic
		NON-AUT	117.00 ± 2.39°	Hydrophobic	34.69 ± 1.09°	Hydrophilic
**Medium 4**	5.5	AUT	89.33 ± 2.87°	Hydrophilic	32.64 ± 0.71°	Hydrophilic
		NON-AUT	87.89 ± 2.10°	Hydrophilic	39.67 ± 2.08°	Hydrophilic
	7.0	AUT	97.41 ± 0.84°	Hydrophobic	41.88 ± 2.00°	Hydrophilic
		NON-AUT	91.32 ± 1.45°	Hydrophobic	33.56 ± 4.24°	Hydrophilic
	8.5	AUT	90.19 ± 2.08°	Hydrophobic	51.40 ± 0.20°	Hydrophilic
		NON-AUT	127.80 ± 3.45°	Hydrophobic	51.84 ± 2.11°	Hydrophilic

**Table 3:** Hydrophilicity or hydrophobicity of the surfaces of the autoclaved (AUT) and non-autoclaved (NON-AUT) micellar membranes of *P. ostreatus* obtained in 1 and 4 according to the measured contact angles.

Medium	pH	Sample	Top Side of the Membrane		Bottom Side of the Membrane	
			Average Contact Angle	Hydrophilic or Hydrophobic Surface	Average Contact Angle	Hydrophilic or Hydrophobic Surface
**Medium 1**	5.5	AUT	89.89 ± 0.88°	Hydrophilic	49.77 ± 3.62°	Hydrophilic
		NON-AUT	89.35 ± 1.04°	Hydrophilic	48.70 ± 1.90°	Hydrophilic
	7.0	AUT	49.48 ± 0.98°	Hydrophilic	71.78 ± 1.75°	Hydrophilic
		NON-AUT	64.30 ± 4.16°	Hydrophilic	62.47 ± 1.95°	Hydrophilic
	8.5	AUT	84.52 ± 2.05°	Hydrophilic	39.52 ± 0.31°	Hydrophilic
		NON-AUT	87.38 ± 2.74°	Hydrophilic	63.33 ± 0.64°	Hydrophilic
**Medium 4**	5.5	AUT	75.43 ± 5.36°	Hydrophilic	77.55 ± 3.16°	Hydrophilic
		NON-AUT	65.29 ± 2.48°	Hydrophilic	70.32 ± 3.66°	Hydrophilic
	7.0	AUT	67.28 ± 4.13°	Hydrophilic	46.60 ± 3.00°	Hydrophilic
		NON-AUT	64.51 ± 3.13°	Hydrophilic	44.61 ± 4.76°	Hydrophilic
	8.5	AUT	55.66 ± 0.21°	Hydrophilic	45.19 ± 4.82°	Hydrophilic
		NON-AUT	68.81 ± 8.55°	Hydrophilic	54.91 ± 0.41°	Hydrophilic

**Table 4:** Measured average diameters of hyphae and pores of AUT and NON-AUT *G. lucidum* and *P. ostreatus* micellar membranes, obtained under optimal growth conditions.

Sample		Average Diameters of Hyphae (min.–max Diameter) (nm)	Average Diameters of Pores (min.–max Diameter) (µm)
*G. lucidum*	AUT membrane	516.75 (126.0–1170.0)	22.14 (13.0–39.1)
	NON-AUT membrane	425.02 (78.1–922.0)	40.30 (28.3–48.1)
*P. ostreatus*	AUT membrane	1150.71 (384.0–2880.0)	/
	NON-AUT membrane	1310.80 (213.0–3030.0)	25.08 (10.5–41.6)

**Table 5:** Kinetic parameters of in vitro release of incorporated MPE (14.5 mg/mL) and CIP (2 mg/mL) from *G. lucidum* and *P. ostreatus* membranes fitted to four mathematical models (k, the release constant; *n*, the release exponent; R^2^, coefficient of determination).

Model	Zero-Order		First-Order		Higuchi		Korsmeyer-Peppas			Release Mechanism
	R^2^	k_0_	R^2^	k_1_	R^2^	k_H_	R^2^	k_KP_	*n*	
MPE-loaded *G. lucidum* membrane	0.761	0.016	0.963	0.003	0.837	1.566	0.971	6.347	0.324	Fickian diffusion mechanism
MPE-loaded *P. ostreatus* membrane	0.645	0.010	0.930	0.005	0.507	1.252	0.944	9.435	0.244	Fickian diffusion mechanism
CIP-loaded *G. lucidum* membrane	0.121	0.004	0.999	0.034	-	-	0.838	15.894	0.093	Fickian diffusion mechanism
CIP-loaded *P. ostreatus* membrane	0.078	0.004	0.999	0.060	-	-	0.906	26.728	0.053	Fickian diffusion mechanism

**Table 6:** Total weight loss of control and with MPE- or CIP-functionalized micellar membranes of *G. lucidum* and *P. ostreatus*.

Membrane	Total Weight Loss (%)
Control *G. lucidum* membrane	71.7
MPE-loaded *G. lucidum* membrane	66.1
CIP-loaded *G. lucidum* membrane	64.3
Control *P. ostreatus* membrane	71.3
MPE-loaded *P. ostreatus* membrane	70.4
CIP-loaded *P. ostreatus* membrane	66.7

**Table 7:** Antimicrobial activity of control and functionalized micellar membranes of *G. lucidum* and *P. ostreatus* with MPE, as well as free MPE and CIP, expressed as the zone of growth inhibition (mm).

Bacterium	Inhibition Zone (mm)							
	Control *G. lucidum* Membrane	MPE-Loaded *G. lucidum* Membrane	CIP-Loaded *G. lucidum* Membrane	Control *P. ostreatus* Membrane	MPE-Loaded *P. ostreatus* Membrane	CIP-Loaded *P. ostreatus* Membrane	MPE	CIP
*E. coli*	/	11.7 ± 0.3	41.3 ± 1.2	/	11.3 ± 0.6	41.0 ± 1.0	13.3 ± 0.6	43.0 ± 1.0
*S. aureus*	/	10.3 ± 0.6	40.3 ± 0.6	/	10.0 ± 0.0	40.7 ± 0.6	10.7 ± 0.6	41.3 ± 0.6

## Data Availability

Data are contained within the article.

## Supplementary Materials

The following supporting information can be downloaded at: https://www.mdpi.com/article/10.3390/polym17172334/s1, Table S1: The presence of functional groups in the tested autoclaved (AUT) and non-autoclaved (NON-AUT) G. lucidum membranes, obtained in media 2 and 4 at three different pH values, treated with autoclaving or without treatment; Table S2: The presence of functional groups in the tested autoclaved (AUT) and non-autoclaved (NON-AUT) P. ostreatus membranes, obtained in media 1 and 4 at three different pH values, treated with autoclaving or without treatment.

## Abbreviations

The following abbreviations are used in this manuscript:

MPE	Mango peel extract
IE	Incorporation efficiency
LC	Loading capacity
UAE	Ultrasound-assisted extraction
CIP	Ciprofloxacin
FTIR	Fourier-transform infrared spectroscopy
SEM	Scanning electron microscopy
TGA/DSC	Thermogravimetric analysis/differential scanning calorimetry
CR	Cumulative release
AUT	Autoclaved
NON-AUT	Non-autoclaved
PDA	Potato dextrose agar
PBS	Phosphate-buffered saline
PLGA	Poly(lactic-co-glycolic) acid
PCL	Poly(ε-caprolactone)
