# Supplementary material for: Functionalized Micellar Membranes from Medicinal Mushrooms as Promising Self-Growing Bioscaffolds
Source: Polymers (Basel). 2025 Aug 28;17(17):2334. doi: 10.3390/polym17172334 (PMC12431496; doi:10.3390/polym17172334)
Supplement: Supplementary file 1 [file polymers-17-02334-s001.zip › polymers-3775892-supplementary.pdf]

Supplementary Materials

Table S1. The presence of functional groups in the tested autoclaved (AUT) and non-autoclaved (NON-AUT) *G. lucidum* membranes, obtained in media 2 and 4 at three different pH values, treated with autoclaving or without treatment.

**Functional group**	**Wavenumber (cm^{-1})**	**Sample**												**Mycelium components**
		pH=5.5		pH=7.0		pH=8.5		pH=5.5		pH=7.0		pH=8.5		
		Medium 2						Medium 4						
		AUT	NON-AUT	AUT	NON-AUT	AUT	NON-AUT	AUT	NON-AUT	AUT	NON-AUT	AUT	NON-AUT	
**O-H stretching**	3570-3200	+	+	+	+	+	+	+	+	+	+	+	+	Polysaccharides
**CH_2 asymmetric stretching**	2935-2915	+	+	+	+	+	+	+	+	+	+	+	+	Lipids
**CH_2 symmetric stretching**	2865-2845	+	+	+	+	+	+	-	+	+	+	+	+	Lipids
**Amide I**	1700-1600	+	+	+	+	+	+	+	+	+	+	+	+	Proteins
**Amide II**	1600-1500	+	+	-	+	+	+	+	+	-	+	-	+	Proteins
**C-H bending**	1370-1350	+	+	-	+	+	+	+	+	-	+	+	+	Chitin
**Amid III**	1250-1350	-	+	+	+	-	+	-	-	-	-	-	+	Proteins
**PO_2^- asymmetric stretching**	1255-1245	-	+	+	+	+	+	+	+	+	+	+	+	Nucleic acids
**C-OH stretching**	1200-1020	-	+	+	+	+	+	+	+	+	+	+	+	Polysaccharides

**C-O stretching**	1085-1050	+	+	+	+	+	+	+	+	+	+	+	+	Polysaccharides
**C-C stretching**	1300-800	+	+	+	+	+	+	+	+	+	+	+	+	Polysaccharides
**Glucan β-anomer C-H bending**	900-800	-	+	-	-	-	+	-	-	-	+	+	+	Polysaccharides
**Mannan band**	875-800	-	-	-	-	-	+	-	-	+	+	+	+	Polysaccharides

Table S2. The presence of functional groups in the tested autoclaved (AUT) and non-autoclaved (NON-AUT) *P. ostreatus* membranes, obtained in media 1 and 4 at three different pH values, treated with autoclaving or without treatment.

**Functional group**	**Wavenumber (cm⁻¹)**	**Sample**												**Mycelium components**
		pH=5.5		pH=7.0		pH=8.5		pH=5.5		pH=7.0		pH=8.5		
		Medium 1						Medium 4						
		AUT	NON-AUT	AUT	NON-AUT	AUT	NON-AUT	AUT	NON-AUT	AUT	NON-AUT	AUT	NON-AUT	
**O-H stretching**	3570-3200	+	+	+	+	+	+	+	+	+	+	+	+	Polysaccharides
**CH_2 asymmetric stretching**	2935-2915	+	+	+	+	-	+	+	+	+	+	+	+	Lipids
**CH_2 symmetric stretching**	2865-2845	-	-	-	-	-	+	-	-	-	+	+	+	Lipids
**Amide I**	1700-1600	+	+	+	+	+	+	+	+	+	+	+	+	Proteins
**Amide II**	1600-1500	+	+	+	+	+	+	-	+	+	+	+	+	Proteins

**C-H bending**	1370-1350	+	+	+	+	+	+	-	+	+	+	+	+	Chitin
**Amid III**	1250-1350	-	-	-	-	-	-	-	-	-	+	-	-	Proteins
**PO_2^- asymmetric stretching**	1255-1245	+	+	+	+	+	+	+	+	+	+	+	+	Nucleic acids
**C-OH stretching**	1200-1020	+	+	+	+	+	+	+	+	+	+	+	+	Polysaccharides
**C-O stretching**	1085-1050	+	+	+	+	+	+	+	+	+	+	+	+	Polysaccharides
**C-C stretching**	1300-800	+	+	+	+	+	+	+	+	+	+	+	+	Polysaccharides
**Glucan β-anomer C-H bending**	900-800	-	-	-	-	-	-	+	+	+	+	+	+	Polysaccharides
**Mannan band**	875-800	-	+	-	-	-	+	-	-	-	-	-	+	Polysaccharides
